# The Influence of Oxidative Stress Markers in Patients with Ischemic Stroke

**DOI:** 10.3390/biom14091130

**Published:** 2024-09-06

**Authors:** Hanna Pawluk, Agnieszka Tafelska-Kaczmarek, Małgorzata Sopońska, Marta Porzych, Martyna Modrzejewska, Mateusz Pawluk, Natalia Kurhaluk, Halina Tkaczenko, Renata Kołodziejska

**Affiliations:** 1 Department of Medical Biology and Biochemistry, Faculty of Medicine, Collegium Medicum in Bydgoszcz, Nicolaus Copernicus University in Torun, Karlowicza 24, 85-092 Bydgoszcz, Poland; m.soponska@cm.umk.pl (M.S.); m.porzych@o2.pl (M.P.); martyna.modrzejewska@cm.umk.pl (M.M.); pawluk.mateusz23@gmail.com (M.P.); 2 Department of Organic Chemistry, Faculty of Chemistry, Nicolaus Copernicus University, Gagarina 7, 87-100 Torun, Poland; tafel@umk.pl; 3 Institute of Biology, Pomeranian University in Slupsk, Arciszewski 22B, 76-200 Slupsk, Poland; natalia.kurhaluk@apsl.edu.pl (N.K.); halyna.tkachenko@apsl.edu.pl (H.T.)

**Keywords:** oxidative stress, biomarkers, stroke

## Abstract

Stroke is the second leading cause of death worldwide, and its incidence is rising rapidly. Acute ischemic stroke is a subtype of stroke that accounts for the majority of stroke cases and has a high mortality rate. An effective treatment for stroke is to minimize damage to the brain’s neural tissue by restoring blood flow to decreased perfusion areas of the brain. Many reports have concluded that both oxidative stress and excitotoxicity are the main pathological processes associated with ischemic stroke. Current measures to protect the brain against serious damage caused by stroke are insufficient. For this reason, it is important to investigate oxidative and antioxidant strategies to reduce oxidative damage. This review focuses on studies assessing the concentration of oxidative stress biomarkers and the level of antioxidants (enzymatic and non-enzymatic) and their impact on the clinical prognosis of patients after stroke. Mechanisms related to the production of ROS/RNS and the role of oxidative stress in the pathogenesis of ischemic stroke are presented, as well as new therapeutic strategies aimed at reducing the effects of ischemia and reperfusion.

## 1. Introduction

Stroke is the second most common cause of death worldwide, with its prevalence rapidly increasing, particularly in developing nations like China, which is home to over 20% of the world’s population [1]. Acute ischemic stroke (AIS) is a subtype of stroke, accounting for the majority of stroke cases, with a high mortality rate. Treatment for AIS should not be delayed, as even a short delay increases the risk of complications, which can be fatal.

An effective approach to treating stroke is to minimize damage to the brain’s neuronal tissue by restoring blood flow to hypoperfused areas of the brain [2]. The only approved pharmacological intervention is the intravenous administration of thrombolytic recombinant tissue plasminogen activator (rtPA), but this applies only to patients, in whom ischemia occurs within the first 4.5 h. Recanalization with rtPA is quite an effective treatment method, but there is a risk of hemorrhage, which consequently may lead to serious post-stroke complications [3]. In addition to non-invasive stroke treatment, mechanical thrombectomy is currently used, during which the thrombus is removed using a microcatheter. Compared to rtPA treatment, thrombectomy can be performed in a wider time window, within 6 to 24 h after the onset of stroke, in the case of damage to a large vessel [4].

In systemic homeostasis, free radicals are inactivated by endogenous and exogenous antioxidants and do not have a destructive effect. The human body has protective mechanisms, including enzymatic systems (superoxide dismutase SOD, glutathione peroxidase GPx, catalase CAT) and non-enzymatic systems, such as vitamins, coenzyme Q, selenium, and melatonin [5].

The imbalance between the continuous production of reactive oxygen species and their elimination as a result of enzymatic and non-enzymatic neutralization reactions and the action of exogenous antioxidants causes oxidative stress. Many reports have concluded that both oxidative stress and excitotoxicity are the main pathological processes involved in ischemic stroke as well as in many nervous system disorders [6].

It is important to emphasize that current measures to protect the brain against serious damage caused by stroke are insufficient. Therefore, it is crucial to investigate both oxidative and antioxidant strategies to reduce oxidative damage.

This manuscript presents current findings on oxidative stress-related issues in stroke patients. Including studies assessing the concentration of oxidative stress biomarkers and the level of antioxidants (enzymatic and non-enzymatic) and their impact on the clinical prognosis of stroke patients. This review also focuses on the role of oxidative stress in the pathogenesis of ischemic stroke, and new therapeutic strategies to be tested to reduce brain damage associated with both ischemia and reperfusion.

In this review, we also wanted to highlight the role of oxidative stress markers in the ongoing pathological processes leading to brain damage. This will allow us to indicate their role in the diagnosis, treatment, and prognosis of the patient. Describe the complex molecular pathways modulated by reactive oxygen and nitrogen species (ROS/RNS) during hypoxia, ischemia, or reperfusion. A detailed understanding of the stroke etiology will enable the introduction of effective therapy in its treatment. So far, no golden solution for quick and effective diagnosis or prevention has been found. This is an important clinical problem because it affects increasingly younger patients, and therefore the search for antioxidant therapy is fully justified.

## 2. Pathomechanism of Ischemic Stroke

**Figure 1:**
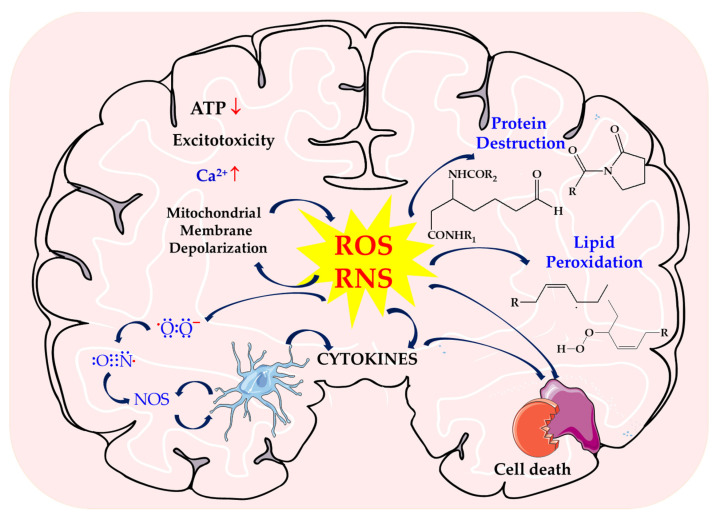
Pathomechanism of ischemic stroke. As a result of hypoxia, there is a deficit of ATP, a decrease in energy, an influx of calcium, and, as a result, mitochondrial failure. Excitotoxicity and ROS/RNS activity stimulate nerve cells, mainly microglia and astrocytes, to secrete inflammatory markers. Increased activity of pro-inflammatory cytokines generates ROS, which are responsible for protein oxidation, peroxidation of polyunsaturated fatty acids, and disruption of redox homeostasis, which ultimately leads to cell death. Abbreviations: ATP—adenosine triphosphate; RNS—reactive nitrogen species; ROS—reactive oxygen species. This figure was created using Servier Medical Art (available at https://smart.servier.com/) (accessed on 12 January 2024).

To fully understand the involvement of ROS/RNS in stroke, the etiology of the disorder caused by hypoxia/ischemia or reperfusion was presented. These processes result in excitotoxicity, oxidative stress, mitochondrial damage, inflammation, energy depletion, and loss of ion homeostasis. They are complex and involve interconnected molecular and cellular mechanisms. As a result of hypoxia, pCO_2_ increases, acidification and ATP decrease and increased oxidative stress causes microvascular damage and the development of inflammation [7,8,9] (Figure 1).

As a result of the ATP deficit, there is a decrease in energy and, consequently, cell death in the area of the brain infarction. The influx of calcium, sodium, and chloride ions into the cell with a simultaneous extracellular increase in K^+^ ions causes depolarization of the cell membrane, which consequently leads to the development of cytotoxic edema. A decrease in ATP reduces the reuptake of glutamate, which leads to neuronal death. Increased activity of neurotransmitter receptors leads to excitotoxicity and the accumulation of Ca^2+^ ions and, as a result, mitochondrial failure and apoptosis [8,10,11].

The development of oxidative stress and increased production of ROS/RNS in neurons prevents the flow of calcium ions across the membrane and activates catabolic enzymes [12].

Excitotoxicity and ROS activity stimulate nerve cells, mainly microglia and astrocytes, to secrete inflammatory markers (interleukins and matrix metalloproteinases (MMP)) [13,14].

Infiltration of immune cells can damage the ischemic brain by producing NO, ROS, and prostanoids, which may lead to increased blood–brain barrier (BBB) permeability and secondary complications [9,13,15].

Injured neurons, damage-associated release molecular patterns (DAMPs) that induce toll-like receptors (TLs) [16,17]. TLR activation enhances the expression of the inactive inflammasome (NLRP3) and pro-IL-1 via signal 1 (NF-κB) which ultimately leads to cytokine production [18,19].

Increased activity of pro-inflammatory cytokines generates ROS, which is responsible for protein oxidation, peroxidation of polyunsaturated fatty acids, and disruption of redox homeostasis within the cell [8,20,21].

As a result of their action, intracellular damage also occurs, i.e., inactivation of enzymes, structural changes in carbohydrate molecules, and ultimately apoptosis [6].

## 3. The Role of Mitochondria in Stroke

Regarding the pathology of stroke, it is believed to be caused by a blockage of a main artery in the brain, which usually leads to cell death. Ischemia causes depolarization of the mitochondrial membrane potential, decreased ATP production, recruitment of Parkin, accumulation of PINK1, overproduction of ROS/RNS, increase in calcium, and opening of the mitochondrial permeability transition pore, which ultimately leads to neuronal cell death [22].

After ischemia, glucose oxidation is significantly reduced, both due to lower tissue energy demand and limitations in mitochondrial pyruvate oxidation. During this process, secondary mitochondrial function deteriorates, and any mitochondrial defects have a very adverse effect on the central nervous system (CNS) [22]. Damage to these organelles may also contribute to progressive cell loss. Mitochondrial release of many apoptogenic proteins has been observed in the brain after ischemia and after ischemia, which occurs mainly in neurons [23].

Mitochondria are very important in this multistep and complicated process. They are centers where cellular calcium signaling occurs [24].

These organelles possess enzyme complexes necessary to maintain the cell’s energy demand and metabolic homeostasis [25].

Function, including managing redox status, modulating Ca^2+^ homeostasis, producing ATP, and regulating the response to cellular and environmental stresses.

The survival of cells, especially neurons, which are exposed to ischemia and hypoxia during a stroke, depends on their integrity and functionality. Alterations in their permeability are a significant factor contributing to tissue damage, especially when induced by short periods of transient focal ischemia. It was also proven that morphological changes in ischemia-sensitive mitochondria were reversible in the case of moderate and mild brain damage [26].

Moreover, cells subjected to oxidative stress or dying release mitochondria, which act as warning signals of impending danger. However, the mechanisms underlying the release and uptake of these structures are still unclear. Many studies suggest that the transfer of mitochondria from one cell to another is a protective mechanism responsible for rescuing damaged cells from mitochondrial dysfunction in response to stress [25,26,27,28,29].

This proves that mitochondrial transfer can lead to a metabolic dialogue between healthy and damaged cells. The released mitochondria can be taken up and re-programmed by recipients, which can activate signals necessary for cell survival. For this reason, it is expected that initiating such a transfer of mitochondria between cells will constitute a new therapeutic approach in the case of many mitochondrial diseases and disorders. It may also involve a stroke [22].

## 4. Sources of Reactive Oxygen and Nitrogen Species

The brain is a highly metabolically active organ, although it does not perform any mechanical or secretory work and requires a constant supply of oxygen and glucose. Temporary or permanent interruption of blood flow in the brain, reperfusion leads to disruption of oxidative–antioxidant homeostasis, i.e., oxidative stress. The lack of this balance results in excessive production of ROS/RNS, which leads to brain damage after a stroke and permanent or reversible neurological deficits.

ROS/RNS in physiological amounts serves as mediators and regulators, ensuring proper cellular functions: growth, proliferation, differentiation, and apoptosis. They participate in various processes, including muscle contractions, hormone secretion, functioning of the defense system, and regulation of vascular tone. They have a direct destructive effect on the body when they are produced in excess. ROS/RNS are involved in many pathological processes, damaging proteins, lipids, carbohydrates, and nucleic acids; therefore, they are responsible for the pathogenesis of many diseases, including ischemic stroke [30].

**Figure 2:**
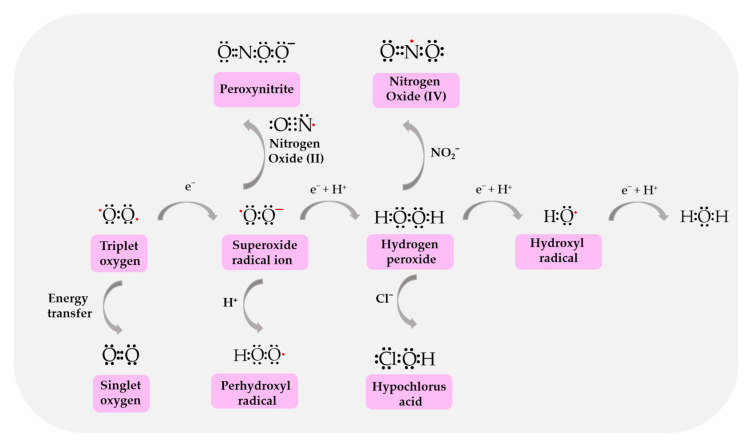
Reactive species of oxygen and nitrogen. The ROS/RNS group includes superoxide anion radical (O_2_^•−^), hydroxyl radical (OH^•^), hydroperoxide radical (HO_2_^•^), singlet/triplet oxygen; nitrogen free radicals: nitric oxide (II) (NO), nitrogen oxide (IV) (NO_2_); non-radical forms: hydrogen peroxide (H_2_O_2_), peroxynitrite ion (OONO^−^), hypochlorous acid (HOCl).

ROS/RNS have one unpaired electron in their valence shell, which determines their high reactivity. The ROS/RNS group that mediates the toxic effect of triplet oxygen (oxygen in the ground state) includes superoxide anion radical (O_2_^•−^), hydroxyl radical (OH^•^), hydroperoxide radical (HO_2_^•^), singlet oxygen and free nitrogen radicals: nitric oxide (NO), dioxide nitrogen (NO_2_) and non-radical forms: hydrogen peroxide (H_2_O_2_), peroxynitrous acid (OONOH), chloric acid (I) (hypochlorous acid, HClO) and hypothiocyanate acid (HOSCN) (Figure 2). Iron–oxygen complexes such as the ferryl radical (Fe(IV)=O^•+^) and the perferryl radical (Fe(III)=O_2_^•+^) are also considered to be reactive oxygen species. The reaction of free radicals with organic compounds produces, among others, alkoxy radicals (RO^•^), peroxide radicals (ROO^•^), semiquinone radicals (H-Ch^•^) and semiquinone anion radicals (Ch^•−^), as well as related non-radical forms such as iodic acid (I) (hypoiodous acid, HIO) or bromic acid (I) (hypobromous acid, HBrO) [31].

Neurons are particularly sensitive to stress caused by ROS/RNS overproduction due to their relatively low levels of antioxidants, high levels of pro-oxidants (iron), unsaturated fatty acids, and high rates of oxidative metabolism compared to other cells. During ischemia/hypoxia, glucose and oxygen deficiency impair energy metabolism, initiating a cascade of damage caused by oxidative stress. Mitochondria, xanthine oxidoreductase, and NADPH oxidase are mainly responsible for the production of reactive oxygen and nitrogen species. Myeloperoxidase, ferroptosis, and stress of the endoplasmic reticulum are also sources of free radicals and reactive non-radical species.

Figure 2 shows the most important mechanisms for generating reactive species, which are discussed in detail in the following subsections.

### 4.1. ROS/RNS Production by Mitochondria

Physiologically, the primary ROS/RNS production in neurons is constitutively generated by mitochondrial metabolism, which is higher than in other cells due to the need to maintain the activity of neuronal circuits and synaptic transmission [32].

**Figure 3:**
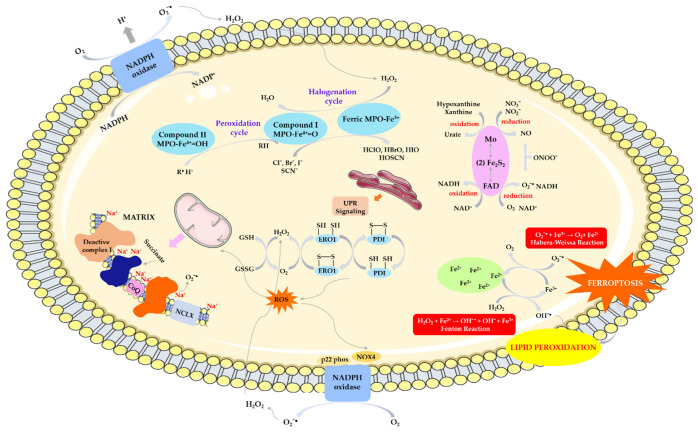
Sources of reactive oxygen. (**A**) ROS/RNS generation by mitochondria. Deactivation of complex I results in activation of the mitochondrial Ca^2+^/Na^+^ antiporter (NCLX), reducing the fluidity of the inner mitochondrial membrane (IMM). The decrease in IMM fluidity favors the formation of superoxide anion. (**B**) ROS generation by NAD(P)H oxidase: NAD(P)H oxidase transfers electrons from NADPH to oxygen while producing superoxide anion: 2O_2_ + NADPH → 2O_2_^−^ + NADP + H^+^. (**C**) ROS generation by ferroptosis: Ferroptosis results in iron overload and the lethal accumulation of lipid peroxides and reactive oxygen species (ROS) in the cell. In the Fenton reaction, in the presence of Fe^2+^, a hydroxyl radical is formed: H_2_O_2_ + Fe^2+^ → OH^−•^ + OH^−^ + Fe^3+^, which in the subsequent reaction oxidizes lipids. (**D**) ROS/RNS generation by Xanthine oxidase (XO): Xanthine oxidase oxidizes xanthine and hypoxanthine, providing two electrons to the molybdenum cofactor. Electrons travel through iron–sulfide residues to FAD, which transfers two or one electron to oxygen, resulting in the formation of hydrogen peroxide or superoxide anion radical, respectively. (**E**) ROS generation by Myeloperoxidase (MPO): MPO in the presence of hydrogen peroxide catalyzes one- or two-electron oxidation reactions and transiently produces many redox heme iron species. MPO oxidizes Cl^−^, Br^−^, I^−^, SCN^−^ ions (halogenation cycle) to form hypohalous acids (HOBr, HOCl, HOI) and HOSCN and small molecule substrates in the peroxidase cycle. (**F**) ROS generated by endoplasmic reticulum (ER) stress: The unfolded protein triggers ROS. In the newly created proteins, thiol groups are oxidized, and disulfide bridges are formed. These reactions are catalyzed by ERO1 oxidoreductase and PDI isomerase, accompanied by electron transfer via FAD to molecular oxygen and the formation of hydrogen peroxide. PDI, ERO1 also catalyzes the conversion of glutathione (GSH) to glutathione disulfide (GSSG). Abbreviations: CoQ—coenzyme Q (Cytochrome c—oxidoreductase (complex III)); ERO1—ER oxidoreductin 1; GSH—glutathione; GSSG—glutathione disulfide; MPO—myeloperoxidase; NAD—nicotinamide adenine dinucleotide; NADP—nicotinamide adenine dinucleotide phosphate; NAD(P)H oxidase—nicotinamide adenine dinucleotide phosphate oxidase; NCLX—Ca^2+^/Na^+^ antiporter; PDI—protein disulfide isomerase; UPR—unfolded protein response. This figure was created using Servier Medical Art (available at https://smart.servier.com/) (accessed on 12 January 2024).

The mitochondria, and more specifically the mitochondrial respiratory chain, is one of the main sites of free radical formation in hypoxic conditions. In the state of hypoxia, as a result of feedback, the rate of electron flow through the respiratory chain decreases, leading to an increase in the concentration of superoxide anion radical, mainly through complex III of the respiratory chain. The mechanism of hypoxic ROS production has recently been discovered [33]. During the first minutes in a state of hypoxia, conformational changes in complex I occur, as a result of which it enters a dormant state in which it cannot perform its enzymatic activity [34,35]. Deactivation of complex I causes acidification of the mitochondrial matrix, which results in the precipitation of calcium phosphate, which partially dissolves and releases free calcium. As a result, the mitochondrial Ca^2+^/Na^+^ antiporter (NCLX) is activated, which promotes the entry of sodium ions into the mitochondria, causing a decrease in the fluidity of the inner mitochondrial membrane (IMM). The decrease in IMM fluidity reduces the transfer of ubiquinol between complexes II and III, which promotes the formation of superoxide anion [36] —Figure 3. The resulting O_2_^•−^ can generate a hydroperoxy radical (HO_2_^•^) and also dismutate into hydrogen peroxide in reaction with superoxide dismutase (MnSOD—located inside the matrix and Cu/ZnSOD—associated with the outer membrane of the mitochondria).

During hypoxia, the activity of mitochondrial nitric oxide synthase (mtNOS, EC 1.14.13.39) also increases, leading to an increase in NO concentration, which inhibits the activity of cytochrome oxidase—complex IV (EC 1.9.3.1). This inhibition leads to a partial increase in O_2_ pressure in the mitochondrial environment and thus increased production of superoxide anion. Nitric oxide reacts with superoxide anion to form peroxynitrite (ONOO^−^), which decomposes to form strongly oxidizing products: NO_2_ and OH^•^. Nitrogen dioxide oxidizes and nitrates many important biomolecules, including polyunsaturated fatty acids, single amino acids, and whole proteins. Peroxynitrite inhibits complexes I, II, and IV of the respiratory chain, ATP synthase, aconitase, creatinine kinase, and MnSOD. The action of reactive oxygen and nitrogen species also affects calcium homeostasis, there is an increased outflow of Ca^2+^ from the matrix through the mitochondrial megachannel PTP (permeability transition pore). Alternatively, OONO^−^ may stimulate calcium efflux through the Na^+^/Ca^2+^ antiporter [37].

### 4.2. ROS Production by Xanthine Oxidoreductase

Reactive oxygen and nitrogen species are also produced by the activation of xanthine oxidoreductase (XOR) in response to oxygen and glucose deprivation. XOR is molybdenum–iron–sulfur flavin hydroxylase, which exists in two forms: xanthine dehydrogenase (XDH) and xanthine oxidase (XO). XDH can be converted to XO irreversibly by proteolysis or reversibly by modification of cysteine residues. The physiological substrates for xanthine oxidase, xanthine, and hypoxanthine, provide two electrons to the molybdenum cofactor, changing the oxidation state of molybdenum from +4 to +6. An oxygen atom is added to the substrate, while electrons travel through iron–sulfide residues to FAD. FAD is reoxidized by transferring two or one electron to oxygen, producing hydrogen peroxide or superoxide anion radicals, respectively. The one-electron reduction of hydrogen peroxide by the Haber–Weiss and Fenton reactions produces the hydroxyl radical, one of the most reactive molecules in biological systems. Under ischemic conditions, xanthine dehydrogenase is converted to xanthine oxidase and hypoxanthine (resulting from the breakdown of ATP) accumulates. Acidic pH and low O_2_ pressure caused by hypoxia reduce the formation of nitric oxide (II) by NO synthase and increase its potential to uncouple and produce superoxide anion. These conditions decrease the affinity of XO for xanthine while increasing the affinity for nitrite, which competes with xanthine at the Mo-co site and can be reduced to NO. Under the same conditions, the amount of superoxide anion formed by XOR is sufficient to react with NO and generate reactive nitrogen species (RNS), especially ONOO^−^. Both free radicals, such as HO^•^ and NO, and non-radical species, such as H_2_O_2_ and ONOO^−^, have oxidizing effects, thus contributing to oxidative stress [38,39]—Figure 3.

### 4.3. ROS Production by NAD(P)H Oxidase

In a cerebral stroke, NAD(P)H oxidase (EC 1.6.3.1) plays an important role in generating superoxides in immune cells. It is a multimeric enzyme complex of proteins from the NOX family associated with the cell membrane, consisting of cytoplasmic subunits (p47phox, p67phox, p40phox, and Rac2). Its action is to transfer electrons from NADPH to oxygen while producing superoxide anion: 2O_2_ + NADPH → 2O_2_^●−^ + NADP + H^+^. Currently, seven NOX members are known in the NADPH family, including NOX1-5 and dioxygenase (DUOX1-2, also known as NOX6-7). Only NOX1, NOX2, NOX4, NOX5 and their cytoplasmic activator proteins are expressed in neurovascular units and produce a small amount of ROS to maintain the normal physiological activity of the central nervous system [40,41]. Excessive ROS production by inflammatory cells during ischemia occurs through several enzyme systems, but NOX is the main superoxide-producing enzyme (Figure 3). In the course of ischemic stroke, increased expression of NOX2 and NOX4 was found in neurons, astrocytes, and microglia, and NOX1 was increased in microglia [42,43]. The way in which NOX is activated during stroke is not entirely clear, but phosphorylation of the p47 phox subunit seems to be crucial [44].

The NOX4 subtype is the most abundant and is considered the most important source of ROS in the central nervous system [45]. Endothelial NOX4 can damage the blood–brain barrier, leading to BBB leakage, while neuronal NOX4 can directly lead to autotoxicity and apoptosis. Moreover, NOX4 is the main source of peroxidation produced by pericytes [46]. Most studies also support the putative protective effect of NOX2 deficiency in ischemic stroke [46,47].

### 4.4. ROS/RNS Production by Myeloperoxidase

Myeloperoxidase (MPO), a component of neutrophil azurophil granules that remains inactive in the absence of H_2_O_2_, may play a potential role in generating oxidative stress. After neutrophil activation, NOX2 can be incorporated into the neutrophil inner membrane, which then leads to a respiratory burst and H_2_O_2_ production due to the dismutation of the superoxygen radical [48]. As hydrogen peroxide levels increase in neutrophils, MPO is able to catalyze a series of one- or two-electron oxidation reactions and transiently produce multiple redox heme iron species. MPO has two main activities: the oxidation of halogens (halogenation cycle) to form hypohalous acids and the peroxidase cycle (Figure 3). In the halogenation cycle, under the influence of hydrogen peroxide, the native form (Fe(III))-MPO is transformed into the reactive compound I MPO, i.e., the ferryl radical ([Fe(IV=O)^●+]^). Compound I can then oxidize halide/pseudohalide anions (Br^−^, Cl^−^, I^−^ and SCN^−^) to create many hypohalous acids (HOBr, HOCl, HOI, and HOSCN) [49]. HOCl increases toxicity in neuronal cells and astrocytes has high diffusivity and oxidative activity and therefore mediates BBB damage [50]. Moreover, HOCl itself can exacerbate oxidative stress, promote the translocation of p67(phox) and p47(phox) NAD(P)H oxidase, and mediate the production of superoxide, peroxynitrite, and oxidized eNOS dimer in endothelial cells [51].

In the peroxidase cycle, various small-molecule substrates can be oxidized, including xenobiotics, endobiotics, NO, and nitrites, resulting in compound II MPO ([Fe(IV=O]) and diffusive radicals of these substrates (R^•^) [52].

### 4.5. ROS Production by Ferroptosis

In addition to classical pathways, new mechanisms involved in the production of oxidative species have also been identified, including ferroptosis. Normal cell functions are inextricably linked to the presence of iron, therefore maintaining iron homeostasis in the brain is particularly important for cell survival [53]. Ferroptosis is an iron-dependent, regulated cell death that differs morphologically and biochemically from other programmed cell deaths such as apoptosis and necroptosis [54]. The most important biochemical features of ferroptosis are iron overload and the lethal accumulation of lipid peroxides and reactive oxygen species (ROS) in the cell [55], which can produce large numbers of alkyl oxygen radicals, leading to fatal damage and disorganization of the cell membrane [56].

The ferroptosis defense system mainly includes systems dependent on glutathione peroxidase-4 GPX4 (systemXC-GSH-GPX4 axis) and systems independent of it (such as the NAD(P)H-FSP1 (ferroptosis suppressor protein 1)-CoQ (coenzyme Q10) axis, DHFR (dihydrofolate reductase)-BH_4_ (Tetrahydrobiopterin)-GCH1 (GTP cyclohydrolase-1) axis, DHODH dihydroorotate dehydrogenase/CoQ pathway, p62/Keap1 (KELCH-ECH-related protein 1)/Nrf2 (erythroid factor 2-related factor nuclear 2)) [56,57].

The occurrence of ferroptosis requires the synthesis of polyunsaturated fatty acids (PUFA) via lysophosphatidylcholine acyltransferase 3 (LPCAT3) and acyl-CoA synthetase 4 (ACSL4) and PUFA peroxidation via lipoxygenase (ALOX) and cytochrome P450 oxidoreductase (POR) [58].

Intracellular labile iron (Fe^2+^) can generate large amounts of ROS via the Fenton reaction, providing raw materials for lipid peroxidation. It is also a cofactor of lipid peroxidases (ALOXs and POR) and determines the activity of these enzymes. Furthermore, ferroptosis induced by various stimuli is ultimately associated with decreased cellular antioxidant capacity and iron-dependent ROS accumulation (Figure 3). Therefore, ferroptosis is closely associated with oxidative stress [59].

### 4.6. ROS Production by Endoplasmic Reticulum

Endoplasmic reticulum (ER) stress may also participate in the generation of reactive species. The ER contributes to the production and folding of approximately one-third of cellular proteins and is therefore inextricably linked to the maintenance of cellular homeostasis and the fine balance between health and disease. It functions as a protein synthesis factory, and participates in calcium storage and regulation, lipid synthesis and storage, and glucose metabolism [60]. ER stress is defined as a process in which an imbalance is observed between the demand for protein folding and the ability of the ER to fold proteins. Changes in the folding pathway led to the accumulation of misfolded and unfolded proteins, in the ER lumen, which disrupts cellular homeostasis and initiates the unfolded protein response (UPR) [61,62]. The UPR triggers ROS production, and ROS in turn can promote ER stress [63].

In newly created proteins in the ER, thiol groups are oxidized, and disulfide bridges are formed between cysteine molecules. These reactions are catalyzed by ERO1 oxidoreductase and PDI isomerase, accompanied by electron transfer via FAD to molecular oxygen and the formation of hydrogen peroxide. In addition to PDI oxidation, ERO1 also catalyzes the conversion of glutathione (GSH) to glutathione disulfide (GSSG). The accumulation of both H_2_O_2_ and oxidized glutathione causes ER stress (Figure 3). Moreover, the ratio between GSH and GSSG is an important marker of the redox state in the ER lumen. PDI is also involved in H_2_O_2_ production through interconnections with NOX1 and NOX4 [64,65,66]. Additionally, NOX4 also participates in the production of superoxide anion and hydrogen peroxide in the transmembrane ER.

The multienzyme microsomal monooxygenase (MMO) system, consisting of multiple types of cytochrome P450, NADPH-P450 reductase (NPR), and cytochrome b5, is yet another site of ROS production in the ER [67]. MMO catalyzes the oxidation of hydrophobic exogenous compounds and some endogenous substrates, resulting in the formation of superoxide radicals and H_2_O_2_ [68,69]. Furthermore, in the early stages of ER stress, oxidative stress triggers the release of calcium from the ER, promoting its uptake by mitochondria. This increase in calcium concentration enhances metabolic activity and ROS production within the mitochondria. Through feedback mechanisms, calcium ions additionally increase the sensitivity of calcium channels. In earlier stages of ER stress conditions, oxidative stress forces calcium out of the ER and causes reuptake by mitochondria. Therefore, increasing the concentration of calcium increases metabolic activities and ROS generation in mitochondria. Via feedback mechanisms, calcium ions further enhance the sensitivity of calcium channels [70,71].

## 5. Oxidative Potential of the Cell in Stroke

In acute ischemic stroke (AIS) increased production of free radicals, and ROS in tissue and plasma occurs. The balance between ROS and antioxidant activity is disturbed and shifted toward free radicals causing oxidative stress, that potentially leads to damage to cellular lipids, proteins, and DNA. Antioxidants reduce the severity of oxidative stress, either by forming less active radicals or by suppressing harmful free radical chain reactions on macromolecules through oxidation [72,73]. In stroke patients, the antioxidant defense system has been studied concerning enzymes, including superoxide dismutase, catalase, and glutathione peroxidase, and nonenzymatic antioxidants such as ascorbic acid, α-tocopherol, carotenoids, and uric acid [74,75,76,77,78,79]. However, determination of total antioxidant capacity (TAC) in serum or plasma provides more relevant information about the antioxidant status than that obtained from measuring concentrations of individual antioxidant compounds [80,81]. Additionally, TAC may demonstrate the synergistic effects between different antioxidants [82,83,84,85].

The measurement of total antioxidant capacity (TAC) is commonly employed in the evaluation of the antioxidant status in human samples that are linked to various diseases. The TAC assessment demonstrates the overall ability of the body to combat oxidative stress through the production of antioxidant compounds [86]. Total antioxidant capacity (TAC) is another, besides malondialdehyde (MDA), 4-hydroxynon-2-enal (4-HNE), thiobarbituric acid reactive substances (TBARs), 8-hydroxy-2-deoxyguanosine (8-oHdG) biomarker to estimate the total oxidative stress in stroke patients [87]. In the case of TAC, similarly to MDA, contradictory results can be observed in patients with AIS. Menon et al. have found TAC significantly lower in stroke patients than in controls, proving that higher oxidant levels in the system lead to reduced TAC [88]. Similar results have been obtained in several other research [77,78,79,89,90,91,92]. Moreover, Ghonimi’s group found that TAC levels were much lower in AIS patients than in controls, while being lower in patients with large-vessel cerebral infarction (LVD) than in those with small-vessel infarction (SVD) [93]. They concluded that oxidant/antioxidant imbalance is related to the different pathogenesis in the two major subtypes of non-cardioembolic stroke, and this may explain the differences in TAC levels between LVD and SVD infarctions.

On the other hand, a higher circulating TAC level in ischemic stroke patients than in healthy control subjects was established by Demir’s group [94]. The study involved diabetic and nondiabetic acute stroke patients and found that TAC levels were significantly higher in diabetic patients than in nondiabetic and control cases. TAC levels were also higher in nondiabetic stroke patients compared with controls, but the difference was not significant. In this research, no difference was found between TAC levels in large and small vessel stroke subtypes of diabetic and nondiabetic patients. Gerreth et al. have also found no difference between salivary TAC in both—stroke patients and controls [95]. Similar observations were made by Altamura’s group [96].

Lorente et al. established an association between circulating TAC levels and 30-day mortality after severe ischemic stroke [84]. They found higher serum TAC levels in non-surviving patients than in survivors AIS, therefore serum TAC levels could be used as a prognostic biomarker of mortality. In their study, there is a lack of determination of serum TAC levels in healthy controls.

TAC concentration in human stroke is presented in Table 1.

## 6. Defense Mechanisms in Stroke

The brain is particularly sensitive to the effects of oxidative stress because it consumes over 20% of the oxygen used by the body. It is protected against the harmful effects of ROS through the so-called biochemical barrier that includes a whole set of antioxidant and detoxification mechanisms. Due to their mechanism of action, enzymatic and non-enzymatic antioxidants of exogenous and endogenous origin are distinguished. Endogenous antioxidants mainly include enzymes belonging to the first line of defense such as superoxide dismutase (SOD; E.C.1.15.1.1), catalase (CAT; EC 1.11.1.6) and glutathione peroxidase (GPx; EC 1.11.1.9). Antioxidants belonging to the second line of defense include vitamins A, C, and E, bilirubin, glutathione, uric acid, carnitine, flavonoids, coenzyme Q 10, lipoic acid, dihydrolipoic acid, and melatonin [97].

### 6.1. Enzymatic Defense against Oxidative Stress in Stroke

#### 6.1.1. Antioxidant Enzymes

The primary antioxidant enzyme is SOD, which converts toxic superoxide anion into hydrogen peroxide, which is then broken down by CAT and GPx. SOD is a metalloprotein belonging to the enzymes of the oxidoreductase group. It occurs in all aerobic organisms and takes part in the aerobic cellular metabolism pathways, which provide aerobic living organisms with a source of energy and enable life. Three SOD isomers were identified taking into account the structural characteristics (with separate protein folds, different catalytic metal ions) and the place of occurrence: SOD1 (CuZnSOD, cytosolic, SOD1 gene, chromosome 21q22.11), SOD2 (MnSOD, mitochondrial, SOD2 gene, chromosome 6q25.3), SOD3 (EC-SOD, extracellular, SOD3 gene, chromosome 4q15 [98].

SOD1 is a homodimer composed of subunits with a molecular weight of 16 kDa, and in the active center, it contains copper (Cu^2+^) and zinc (Zn^2+^) ions. Copper is located in the catalytic center, and zinc plays a structural role. It occurs mainly in the cytosol of liver, testicular, and kidney cells, as well as in erythrocytes and cells of the central nervous system (CNS). SOD1 is a protein that is resistant to high temperatures and proteolytic enzymes. SOD2 is a tetramer with manganese ions (Mn^2+^) in the active center. It occurs mainly in mitochondria, although small amounts have also been found in intercellular spaces and peroxisomes. SOD3 is a glycoprotein structured similarly to SOD2, instead of manganese it contains zinc and copper. It occurs in tissues, blood plasma, and body fluids such as lymph, synovial fluid, interstitial fluid, and cerebrospinal fluid. The extracellular SOD isoenzyme is located in the extracellular matrix and in the cell membrane, where it is present in a concentration 20 times higher than in the plasma [99,100,101].

**Figure 4:**
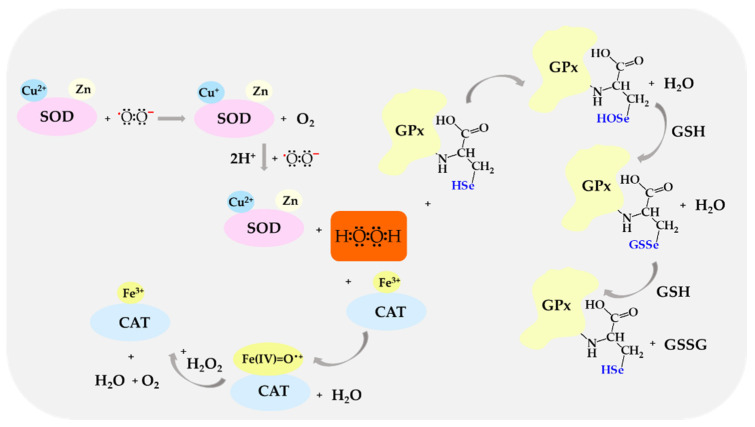
Mechanism of action of antioxidant enzymes. SOD converts superoxide anion into hydrogen peroxide. GPx reduces hydrogen peroxide. The proton donor in the reaction is glutathione. Hydrogen peroxide oxidizes the selenocysteine residue to a selenic acid derivative (RSeOH), which is reduced to selenol (RSeH) in a two-step process. In the first stage, RSeOH and GSH react and GSSeR is formed, which in the next stage is reduced to selenol in the reaction with the second GSH molecule. CAT reduces hydrogen peroxide with the participation of Fe^3+^ iron ions in the heme system. Abbreviations: CAT—catalase; GPx—glutathione peroxidase; GSH—glutathione reduced form; GSSG—glutathione disulfide (glutathione oxidized form); SOD—superoxide dismutase.

SOD catalyzes the disproportion of superoxide into molecular oxygen and hydrogen peroxide (Figure 4). It thus prevents the formation of other reactive oxygen species and their derivatives, interrupting the chain of their production [98,102].

Differences in the activity of SOD isoenzymes in various cells of nervous tissue determine their susceptibility to oxidative stress and neurodegradative diseases. It should be noted that most SOD1 was localized in spinal motor neurons and astrocytes. However, SOD2 was most abundant in brain and spinal cord neurons, and less in astrocytes. Lower amounts of SOD3 compared to body fluids were recorded in the brain, skeletal muscles, liver, and kidney [101].

Catalase is an oxidoreductase found in peroxisomes, mitochondria, and the endoplasmic reticulum. Its high concentration was found in the cytosol of liver cells, kidneys, erythrocytes, bone marrow, and mucous membranes. In the CSN it occurs in the cerebellum and spinal cord. Depending on the concentration of hydrogen peroxide, it has catalase activity, removing hydrogen peroxide, and converting it into water and oxygen. At low H_2_O_2_ concentrations, the peroxidase activity of catalase dominates, and the substrates are hydrogen donor compounds, e.g., ethanol, methanol, phenol, nitrites, quinones, and others. The mechanism of the two-stage hydrogen peroxide disproportionation reaction involving Fe^3+^ iron ions of the heme system is shown in Figure 4 [103].

Glutathione peroxidase, like CAT, reduces hydrogen peroxide. It can also reduce organic peroxides, especially lipid peroxides. Glutathione participates in the reaction as a proton donor. In the CNS, the enzyme is present in the cytoplasm and mitochondrial matrix of nerve cells. Greater activity than in kidney cells was found in micro- and macroglia.

Several GPx isoforms are known, differing in their location and subunit structure. There are GPx1-4, selenoperoxidases with selenocysteine in the active site, cytosolic glutathione peroxidase (GPx1), gastrointestinal peroxidase (GPx2), extracellular peroxidase located in the serum (GPx3) and glutathione peroxidase of phospholipid and cholesterol peroxides (GPx4). In addition, there is Gpx5, which contains cysteine instead of selenocysteine, acts as a secretory enzyme of the epididymis, GPx6, a selenoprotein created by the olfactory epithelium, and the low-activity peroxidases GPx7 and GPx8 [104,105].

The mechanism of action of the enzyme may vary depending on the isoform; in general, as a result of oxidation with hydrogen peroxide, the selenocysteine residue is transformed into a derivative of selenic acid (RSeOH). Selenic acid is then reduced to selenol (RSeH) in a two-step process. The reaction of RSeOH and GSH produces GSSeR and water, which in the next step, together with the second molecule, reduces the intermediate compound back to selenol, releasing GSSG as a by-product (Figure 4) [106,107].

#### 6.1.2. Antioxidant Enzymes in Stroke

The lack of dynamic balance between the production of ROS and the resources of the antioxidant scavenging system leads to supraphysiological ROS concentrations, oxidation–reduction balance disorders, and, consequently, oxidative stress. Under conditions of homeostasis, antioxidant enzymes are synthesized at a constant level and are subject to strict regulation, and under conditions of oxidative stress their activity changes [98].

Data regarding post-stroke changes in the activity of antioxidant enzymes are ambiguous. Some authors reported increased levels of activity of these enzymes [108,109,110,111,112,113] others decreased them [111,114,115,116]. Differences may depend on the methods of preparation and storage of biological samples, measurement methods used, sampling time, and the size and consistency of the study groups.

Žitňanová et al. observed increased SOD activity in hemolysates in patients with acute stroke compared to the control group. The highest activity in the group of patients was on the 7th day after stroke. Similarly, CAT activity was elevated in all patient subgroups compared to controls. However, GPx activity only within 24 h after stroke. Moreover, Western blot analysis showed the highest SOD expression in erythrocyte hemolysates in the group of patients on the 7th day and in the third month after the onset of ischemic stroke compared to the group of people with acute ischemic stroke (24 h). While catalase expression was unchanged in all study groups. Elevated SOD activity may be associated with increased ROS production and enhanced antioxidant defense during AIS. Moreover, the restoration of blood flow and energy homeostasis causes the activation of proteosynthesis of this protein, which is reflected in the increased expression of SOD at later time points compared to the group of patients for whom the test material was collected 24 h after AIS [109].

Increased antioxidant enzyme activity may reduce free radical damage, as reflected in reduced lipid peroxide levels at 7 days and 3 months of follow-up, and provide protection against neurological damage [109]. Moreover, the increase in the activity of antioxidant enzymes is positively correlated with the level of the Nrf2 transcription factor. In response to ROS retention, Nrf2 released from the Keapl-Rnf2 Keap1-Nrf2 protein complex in the cytoplasm is translocated to the cell nucleus where it binds to antioxidant response elements (AREs), activating the transcription of enzymatic antioxidant response genes (SOD, CAT, GPx) [110].

Other authors, assessing the SOD content in erythrocyte hemolysates and plasma in patients with a stroke in the early neurological stage, observed significantly higher activity of this enzyme compared to the control group. While erythrocytes’ GPx activity in all study patients was significantly lower than in the control group. They also found that the group of patients with TIA (transient ischemic attack) showed higher SOD activity and lower GPx activity compared to AIS patients. In these study groups, patients with NIHSS > 10 had higher SOD activity, both in erythrocyte hemolysate and plasma, and lower GPx activity. Patients with a favorable outcome (mRS ≤ 3) assessed by the mRS scale had reduced SOD activity and increased GPx compared to patients with an increased risk (mRS > 3). A positive correlation was observed between the SOD activity of hemolysates and the SOD activity in plasma and the mRS value (r = 0.38; r = 0.30, respectively), and a negative correlation in the case of GPx (r = −0.5) [111]. However, Zimmermann et al. found no relationship between SOD and the NIHSS scale and no differences in enzyme activity in patients immediately after the onset of ischemic stroke and at risk of stroke and in the control group. In the case of GPx, a statistically significantly higher activity of this enzyme in the serum was demonstrated on the first day after stroke compared to the reference group, and a negative correlation between GPx activity and the severity of neurological deficits assessed according to the NIHSS scale in the first 6 h and on the seventh day [112].

In the study by Kolesnichenko et al., plasma and erythrocyte GPx activity of patients with ischemic stroke was also higher in each subgroup of stroke patients mild (NIHSS = 4.6), moderate (NIHSS = 8.9), severe (NIHSS = 15.4) compared to inspection. Statistically significant differences were observed for mean erythrocyte GPx activity in the group of severe stroke patients and the mean GPx activities in plasma of the mild, moderate, and severe stroke patients. An increase in GPx activity protects against reactive oxygen species and peroxidation products, thereby enhancing the redox balance and improving redox regulation. According to the authors, in the case of a severe stroke, it is impossible to survive without the protective reactions of the glutathione system. The most significant changes in the glutathione metabolic system were observed in survived patients with severe IS, with a 4.2-fold increase in GPx activity, which, may be considered as maximal protective reactions [113].

Demirkaya et al. observed lower SOD and GPx levels within 24 h of symptom onset in acute stroke patients compared with controls. SOD and GPx activity were inversely correlated with the size of the infarction and the severity of the neurological deficit assessed according to the NIHSS scale. The levels of SOD and GPx in the stroke groups at the end of the first week increased to the activity values of these enzymes in the control group, suggesting a strengthening of the antioxidant barrier and possible recovery of the patient. The above test results may indicate that antioxidant enzymes may be a good indicator of prognosis and prognostic of the size of infarction [114].

Similarly, Shi et al. observed lower SOD activity in the study group with ischemic stroke compared to the group of healthy people. In their study, patients were divided into four groups depending on the time of stroke onset: group 1 (0:00–05:59); group 2 (06:00–11:59); group 3 (12:00–17:59); group 4 (18:00–23:59). Material for testing in the control group was collected at the same time. Furthermore, all included patients had no previous history of sleep disorders or travel across time zones. The importance of circadian rhythm in SOD levels was observed. Ischemic stroke patients from subgroup 2 showed reduced SOD activity, while the highest activity was found in group 4. A significant reduction in the expression levels of basic helix–loop–helix ARNT Like 1 (BMAL1) and the transcription regulator of circadian clock genes (SIRT1) was also demonstrated in patients with ischemic stroke from group 2. Pearson correlation analysis showed a moderate positive correlation between BMAL1 and SIRT1, as well as between BMAL1 and SOD (r = 0.69; r = 0.73, respectively), indicating that SIRT1-BMAL1 may contribute to the early onset of ischemic stroke by regulating oxidative stress [115].

Moreover, low SOD may be a new risk factor for cognitive impairment and cognitive rehabilitation after stroke. Zhang et al. found that serum SOD levels were significantly lower in patients with cognitive impairment in the early phase and at 3 months after mild AIS than in those without cognitive impairment, accompanied by increased systemic inflammation biomarkers (ESR, CRP, and IL-6). Multivariable analyses showed that low serum SOD levels were associated with a higher risk of cognitive impairment after stroke. High levels of SOD might be one of the protective factors for cognitive rehabilitation after stroke [116].

The value of GPx, CAT, and SOD activity in human stroke is presented in Table 2.

### 6.2. Non-Enzymatic Defense against Oxidative Stress in Stroke

#### 6.2.1. Melatonin

The indoleamine melatonin (MEL, *N*-acetyl-5-methoxytryptamine) is a hormone produced by the pineal gland that has a variety of effects and modulates immune responses, seasonal reproduction, circadian rhythm, and many cellular and molecular targets [117,118]. It also alters metabolism when T cells become malignant. Melatonin is the hormone that plays a role in regulating the sleep-wake cycle through its chronobiotic effects on the suprachiasmatic nucleus of the hypothalamus [119]. Recent studies have suggested that the circadian rhythm, regulated by melatonin, regulates blood pressure and modifies a number of physiological processes, including vascular resistance, metabolic activity, and cardiac abnormalities [120]. Research indicates that the risk of stroke pathology is increased by high blood pressure and other concurrent diseases such as hypertension. Many illnesses of the central nervous system, such as subarachnoid hemorrhage, Parkinson’s disease (PD), Alzheimer’s disease (AD), cognitive decline, and anxiety disorders, are improved by melatonin [121].

Because of its inherent antioxidant properties, melatonin can be regarded as a physiological protective agent against environmental stress. The strongest antioxidant among all plant growth regulators is melatonin, according to research [122,123]. Melatonin is a versatile hormone that influences both the process of growth and development and the ability to withstand the effects of drought. It has the potential to significantly enhance plant cell function by reducing free radicals, enhancing antioxidant activity, enhancing the efficiency of the electron transfer system in mitochondria, controlling metabolite contents, and more [124].

A study conducted on elderly patients for the concentrations of melatonin showed that 6-hydroxymelatonin sulfate is a marker for post-stroke cognitive impairment [125]. Another study found statistically significant differences in the level of melatonin metabolite marked after 24 h in the case of patients with favorable (mRS: 0–2 pts) and unfavorable (mRS: 3–6 pts) functional results in patients after ischemic stroke at the time of admission. The concentration of this metabolite was statistically significantly lower compared to the control group. This may suggest that patients treated with thrombolysis have impaired urinary excretion of the metabolite and poor antioxidant protection. A relationship was found between the assessment of 6-hydroxymelatonin sulfate after 24 h and the severity of the neurological deficit upon admission, which may indicate that melatonin may act as a prognostic factor in the thrombolytic treatment of AIS [5].

An investigation of the antioxidative and anti-inflammatory effects of melatonin on experimental liver damage by radiation showed that melatonin’s antioxidant effect is related to its amphiphilic chemical structure, which permits activity in both lipid and aqueous environments and makes it easier for it to pass through biological barriers. Part of the cytoprotective and anti-inflammatory effects are linked to silent information regulator 1 (SIRT1) pathway activity [124]. Nicotinamide adenine dinucleotide (NAD^+^) is required for the action of a type of histone deacetylase known as SIRT1 [126]. A study was conducted to prove the effects of melatonin on mice, and the results validate the theory that melatonin can prevent stroke in animals by reducing oxidative and inflammatory stress by activating MT2 melatonin receptors [127]. It has been demonstrated that SIRT1 controls canonical TGF-β signaling, which, in turn, regulates tissue fibrosis and fibroblast activation [128]. Cellular organelles and melatonin have a close relationship. Because mitochondria have been shown to contain high levels of melatonin, they may be a suitable target for melatonin. Using the right medication to improve the function of the ER and mitochondria alone or in crosstalk connection can be a useful treatment for stroke, given that stroke causes both mitochondrial and ER dysfunction and that each organelle’s dysfunction exacerbates the other’s effects [129].

It has been discovered that melatonin plays a major part in METH-induced neurotoxicity [130]. Melatonin reduces Aβ-induced neurotoxicity and likely facilitates Aβ clearance via BBB transit, degradation pathways, and glymphatic–lymphatic drainage [131]. Among various endogenous and exogenous neuroprotective agents, such as progesterone and estrogens, melatonin is an excellent neuroprotective agent. Numerous studies have looked into the neuroprotective qualities of MEL in both in vitro and in vivo models of cerebral ischemia. In focal cerebral ischemia injury, MEL increases neurological points, reduces the amount of brain water, and decreases the infarction volume [132]. In experimental stroke models, melatonin has been shown in multiple studies to have neuroprotective effects, as evidenced by reduced infarct and brain edema volume and enhanced neurological function. Stroke incidence has been connected to both abnormal circadian cycles and decreased levels of the circadian hormone melatonin in the blood [133]. A study was subjected to mice with middle cerebral artery occlusion and then reperfused the artery and injected with melatonin to see the effects, after the MCAO, melatonin decreased the infarct amount and enhanced neurological function [134]. Due to melatonin’s anti-inflammatory and antioxidant properties, brain ischemia–reperfusion damage has been demonstrated to benefit from it.

Recent research supporting these findings has shown that pretreatment with melatonin in a permanent (MCAO) model reduced the expression of induced nitric oxide synthase (iNOS) and neuronal nitric oxide synthase (nNOS) while increasing the expression of endothelial nitric oxide synthase (eNOS) [135]. Another study was conducted on patients not eligible for reperfusion therapy and found out that one and three months following treatment, melatonin within 24 h of the stroke’s start, had a positive impact on neurological and functional recovery [136].

Melatonin inhibits neural damage by activating MT1 and MT2 G-protein-coupled receptors, suppressed by Luzindole’s inhibitor. Luzindole attenuates melatonin-induced proliferative effects of iPSCs-derived NSCs, affecting the PI3K/AKT pathway, according to a previous study [137].

Melatonin also possesses anti-apoptotic properties. According to a study conducted on retinal ganglion cells, melatonin can counteract the effects of high intraocular pressure (IOP) on retinal function and reduce the apoptosis of retinal ganglion cells (RGCs) in rats [138]. Another study investigated the relationship between melatonin and regulation of STAT3 phosphorylation. Melatonin inhibited STAT3 phosphorylation in vitro, lowering cellular senescence and apoptosis markers, similar to S3I-201. However, this inhibition was reversed by using recombinant IL-6, which induces STAT3 phosphorylation, in HK-2 cells [139]. Recent studies have found that Chuanzhitongluo (CZTL)—a compound that targets multiple pathways and targets simultaneously—decreases neuronal apoptosis in AIS mice by altering the apoptosis gene profile [140].

Headache, vertigo, and sleepiness are side effects of exogenous melatonin supplements and may mislead patients into thinking that these supplements are typically harmful. However, according to recent studies, taking melatonin in the appropriate dosage has minimal side effects. Seventy-one individuals with primary insomnia participated in a lengthy 26-week randomized controlled study, and no higher rate of side effects was seen than with a placebo. A 3-month trial using 25 mg melatonin to prevent depression after acute coronary syndrome with 252 individuals over 60 years of age did not find any statistically significant differences in the number of side events reported between the melatonin and placebo groups [141].

The complex hormone melatonin, which has a number of physiological uses, has the potential as a therapeutic agent for the treatment of stroke. It possesses neuroprotective, anti-inflammatory, and antioxidant properties in addition to regulating blood pressure, circadian rhythm, and immune system responses. However, it is important to take into account the right dosage and any possible negative effects. A holistic approach to stroke therapy incorporates research on novel drugs like melatonin together with conventional medical interventions and alternative therapies. Further research and clinical trials are required to completely comprehend melatonin’s therapeutic benefits and improve stroke outcomes.

Table 3 shows the value of MEL concentrations in human stroke.

#### 6.2.2. Glutathione

Glutathione (GSH), or γ-L-glutamyl-L-cysteinylglycine, is the main low-molecular-weight thiol compound, commonly found in all eukaryotic, plant, and animal cells. A non-standard amide bond between the amino group of cysteine and the γ-carboxyl group of glutamic acid protects GSH against natural degradation by aminopeptidases. The concentration of intracellular glutathione is specific for a given cell type, and in the brain, it occurs at a level of ~1–3 mM [142].

It is one of the most important antioxidants and buffers redox homeostasis [143]. As a result of oxidation, it turns into glutathione disulfide (GSSG). Glutathione is capable of effectively scavenging a wide range of free radicals, the order of GSH reactivity towards radicals is ^•^OH > ^•^OCH_3_ > ^•^OOH > ^•^OOCCl_3_ > ^•^OOCHCH_2_ > ^•^OOCH_3_. The rate constants range from 2.02 × 10^4^ M^−1^ s^−1^ to the diffusion limit (7.68 × 10^9^ M^−1^ s^−1^) [105].

Moreover, it participates in the detoxification of electrophilic compounds, in the metabolism of leukotrienes and prostaglandins, participates in the reduction of methemoglobin, is a form of cysteine transport, and affects the activity of glycolysis enzymes [144]. The main site of glutathione synthesis is the liver. In the first stage of synthesis, γ-glutamylcysteine is produced. This step is catalyzed by γ-glutamylcysteine synthetase (EC.6.3.2.2.). The activity of this protein is regulated by the principle of negative feedback; the enzyme is inhibited non-allosterically by GSH. In the second step, catalyzed by glutathione synthetase (EC. 6.3.2.3), glutathione is produced [145].

During ischemia/reperfusion, when the antioxidant defense system is unable to counterbalance oxidative stress, endogenous GSH content in the brain is depleted [146,147,148].

Oral administration of exogenous glutathione to rats after cerebral ischemia/reperfusion significantly increases intracerebral GSH levels. Which translates into improvement of the neurological deficit score, infarct size, histologic lesions, proinflammatory cytokines, and BBB disruption [149]. Exogenous GSH exerts a therapeutic effect on ischemic stroke by increasing intrastriatal dopamine (DA) [149].

Similarly, the administration of glutathione ethyl ester at the beginning of reperfusion prevents the decline in mitochondrial complex I activity, which was associated with smaller infarct size and improved neurological outcomes [150].

Transient ischemia causes a decrease in the level of neuronal GSH and its decrease results in a further increase in ROS production, which ultimately leads to the death of neuronal cells and impairment of hippocampal function [106]. GSH may be a risk indicator for the severity of acute stroke. Patients with low total GSH levels (≤1.07 μM) are at increased risk of more severe stroke according to the NIHSS (NIHSS > 10) [151].

Maintaining the neuronal GSH level is therefore an important component in protecting neurons against oxidative stress, and changes in its level after ischemia/reperfusion are the result of adaptive mechanisms [105].

During acute cerebral ischemia caused by hypoperfusion: a short-time (10 min) global and a continuous (3 h) focal ischemia, disturbance of GSH homeostasis was observed. Middle cerebral artery occlusion (MCAO) and bilateral carotid artery occlusion (BCAO) induce a decrease in GSH in brain tissue, while an increase in total GSH is observed in plasma, which may be caused by the activation of GSH synthesis aimed at neutralizing ROS [152].

Namba et al. examined the temporal profile of GSH concentration changes after reperfusion in an animal model. Total GSH concentration was found to fluctuate, decreased in the hippocampus 3 h after the beginning of reperfusion, and showed a maximum decrease at 24 h and an increase at 72 h. This suggests that the endogenous antioxidant GSH requires 72 h to recover in sensitive regions after 20 min of four-vessel occlusion in rats [153].

In mice in which the production of superoxides was reduced by blocking the activity of NADPH oxidase, GSH content was preserved in neurons. GSH levels in neurons were also maintained by treatment with N-acetylcysteine (NAC), a precursor of GSH, which was associated with lower neuronal superoxide signal, oxidative stress, and neuronal death. Increased GSH content in reactive astrocytes and microglia in the hippocampus is observed 3 days after ischemia. Neuronal GSH depletion is therefore both a consequence and a cause of oxidative stress following ischemia/reperfusion. Restoring its level may have a neuroprotective effect [154].

Clinical trials in patients with ischemic stroke showed an increase in GSH levels 48 h after ischemia compared to the control group [155]. Similarly, Zimmermann et al. also found elevated GSH levels in the first days after ischemia, with elevated glutathione peroxidase activity 1 day after stroke. These results suggest that changes in antioxidant capacity are part of the adaptive mechanisms during acute stroke [112].

Based on the conducted research, it can be concluded that GSH plays an important role in protecting neurons against cell damage after transient global cerebral ischemia by mitigating oxidative stress in the hippocampus. It is therefore worth ensuring its appropriate level because the concentration of glutathione in cells is not constant and decreases significantly with age. In order to increase its concentration and strengthen the antioxidant barrier, you should avoid alcohol and smoking, follow an appropriate diet, and increase physical activity.

GSH concentration in human stroke is presented in Table 4.

## 7. Consequences of ROS/RNS Action in Stroke

Stroke is an example of the consequences of disturbed oxidant–antioxidant homeostasis in humans; as a result of obstruction of blood flow and reperfusion, serious neuronal damage occurs. Oxidative stress leading to damage in cells, tissues, and organs involves the formation of ROS/RNS. Overproduction of free radicals can cause direct oxidative damage to cellular macromolecules such as lipids, proteins, and nucleic acids in ischemic tissues, which leads to cell death [87].

### 7.1. Lipid Peroxidation—The Role of MDA in Stroke

**Figure 5:**
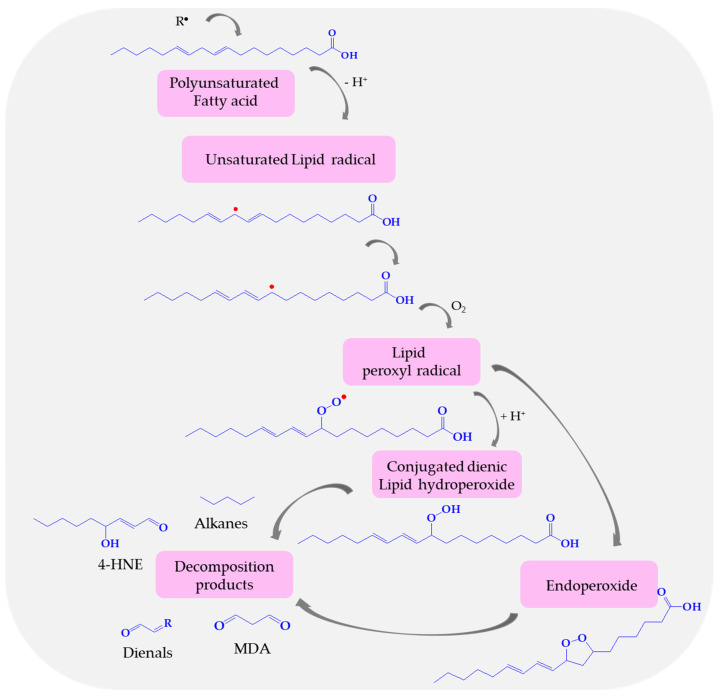
The lipid peroxidation process. ROS attacks lipids containing carbon–carbon double bonds. The hydroxyl radical removes the allyl hydrogen from the fatty acid chain (red dot), leading to the formation of an unsaturated lipid radical, which reacts with molecular oxygen to form a lipid peroxide radical. When the peroxyl radical is located at one of the two ends of the double bond system, the reduction leads to conjugated diene hydroperoxides, while the internal peroxyl radical position results in mono- and/or bicyclic products. Lipid peroxidation products are unstable and decompose into aldehydes—MDA, 4-HNE, and dienals or alkanes. Abbreviations: R^•^—radical; 4-HNE—4-Hydroxynonenal; MDA—malondialdehyde.

Lipid peroxidation is a process under which, mentioned above oxidants—ROS, attack lipids containing carbon–carbon double bonds, especially polyunsaturated fatty acids classified in ω-3 and ω-6 fatty acids (according to the location of the last double bond relative to the terminal methyl end of the molecule), and in consequence, produce lipid free radicals and conjugated dienic hydroperoxides [156]. In the first stage of the lipid peroxidation process, an oxidizing agent, e.g., hydroxyl radical removes the allylic hydrogen from the chain of fatty acid leading to an unsaturated lipid carbon-centered radical (Figure 5). This lipid radical is vulnerable to oxidation and reacts with molecular oxygen, forming a highly unstable lipid peroxyl radical. When the peroxyl radical is located at one of the two ends of the double bond system, the reduction leads to conjugated diene hydroperoxides, while the internal peroxyl radical position results in mono- and/or bicyclic products [87,157]. These toxic and unstable substances easily decompose to secondary products, such as aldehydes—MDA, 4-HNE, and dienals or into alkanes (ethane, pentane). Products of lipid peroxidation, especially aldehydes (MDA, 4-HNE) and TBARs are widely used as biomarkers of oxidative stress [158]. Malondialdehyde appears to be the most mutagenic product of lipid peroxidation, due to its easy diffusion across membranes and covalent modification of protein, whereas 4-HNE is the most toxic [159,160,161]. MDA is capable of irreversible disruption of the enzymes, receptors, and membrane transfer mechanisms [162].

Ischemic stroke patients have shown raised MDA levels compared to the control group [108,111,114,162,163,164,165]. The reason for the increase in MDA in AIS is mainly the conversion of xanthine dehydrogenase to xanthine oxidase and protein kinase activation (due to the activation of phospholipase and protease because of the elevation of cytosolic calcium) [165]. Demirkaya et al. observed increased MDA levels activity significantly correlated with infarct size initial stroke severity assessed NIHSS stroke scale and poor short-term prognosis [114]. Khalili et al. observed that high levels of MDA were associated with increased stroke development, but MDA was not associated with having risk factors for stroke [90]. In this study serum MDA levels were measured within 48 h of stroke onset. Dogan et al. measured the blood MDA levels at admission and again at hours 24, 48, 72, and 96 h, and MDA was constantly higher in patients than in the healthy control group [166]. Additionally, there were no significant changes in MDA levels with time. In the study by Ljubisavljevic et al. plasma MDA values were significantly higher in stroke and TIA patients than in the control group, no differences were observed regarding patients’ clinical severity, clinical outcome, and short-lasting prognosis [111]. On the other hand, Maes’ group has reported no changes in MDA levels (8 h of admission into the hospital) and they explained it by a biphasic response in MDA production [167]. Sharpe et al. found no significant increases in MDA (day of admission) while MDA levels were significantly higher 48 h later [168]. Montaner et al. have stated that different techniques for MDA determinations or stroke severity at baseline might explain the observed differences in the time course of MDA concentration [169].

MDA detection is not restricted to the evaluation of blood samples, but saliva samples can also be used. Salivary MDA with a higher accuracy rate (92%) than serum MDA (81%) differentiates healthy individuals from those with ischemic stroke. Al-Rawi et al. showed that MDA concentrations were significantly higher in the saliva of stroke patients followed by the serum levels [170]. Increased levels of salivary MDA were also noted in patients from stroke risk groups [171].

Moreover, a direct correlation has been found between the increase in MDA and poor functional recovery in acute ischemic stroke [172]. Cognitive impairment is a common consequence of stroke and is associated with poor functional outcomes and a higher risk of recurrent cerebrovascular stroke. Even minor cognitive discrepancies resulting from stroke can distress patients’ quality of life, independent functioning, and occupational capabilities. He et al. have demonstrated that a high serum MDA level at admission is associated with the development of post-stroke cognitive impairment (PSCI) 1 month after AIS and showed significant diagnostic accuracy in discriminating patients with PSCI from patients without cognitive impairment [173]. Similar research conclusions were drawn by El Sayed’s group, who identified the relationship between high serum MDA levels in the first week after AIS, and PSCI after three months [174].

There are also studies that have shown that acute stroke MDA is associated with post-stroke depression (PSD) [175]. Post-stroke depression is a neuropsychiatric complication that frequently occurs after a stroke. The high prevalence of PSD is caused by patient dissatisfaction with the healing process, functional progress, or overall outcome. Depression interferes with the healing process and is a major factor influencing stroke severity, cognitive disorder, and overall mortality. Liu et al. stated that MDA is one of the predictive markers of post-stroke depression, which was supported by Rybka’s study, in which an increased level of MDA was observed in patients diagnosed with severe depression [176,177].

MDA concentration in human stroke is presented in Table 5.

### 7.2. Effects of Nitric Oxide (II)

Nitric oxide (NO) is a one-electron uncharged gas, serving as a signaling molecule crucial in cardiovascular, nervous, and immune systems [178,179].

The principal physiological functions of NO encompass maintaining vascular tone, reducing inflammatory response, balancing thrombotic homeostasis, and regulating cell growth. Nitric oxide synthase (NOS) is the enzyme responsible for NO synthesis. NO is generated via nitrogen oxidation, being endogenously biosynthesized from L-arginine. This amino acid is first converted to N-hydroxyarginine, then to L-citrulline, and finally to NO in the presence of NADPH and BH_4_ as cofactors [180,181].

There are three types of NO synthases (NOS) produced during stroke: inducible NOS (iNOS) synthesizing NO in immune and cardiovascular cells, neuronal NOS (nNOS) synthesizing NO in neural tissues of the central and peripheral nervous system, and endothelial NOS (eNOS) synthesizing NO in blood vessels, regulating vascular function. eNOS and nNOS generate low levels of NO, are constitutively expressed, and are dependent on the Ca^2+^ and calmodulin (CaM) complex, whereas iNOS is expressed in different cell types, for example, macrophages, astrocytes, and microglia, and is calcium-independent. Of these three isoforms, only eNOS plays a neuroprotective role in acute ischemic stroke, while the toxic effects of NO produced by iNOS and nNOS are mainly due to the production of nitrates and the release of free radicals, which directly damage mitochondrial enzymes and genetic materials [182,183,184].

**Figure 6:**
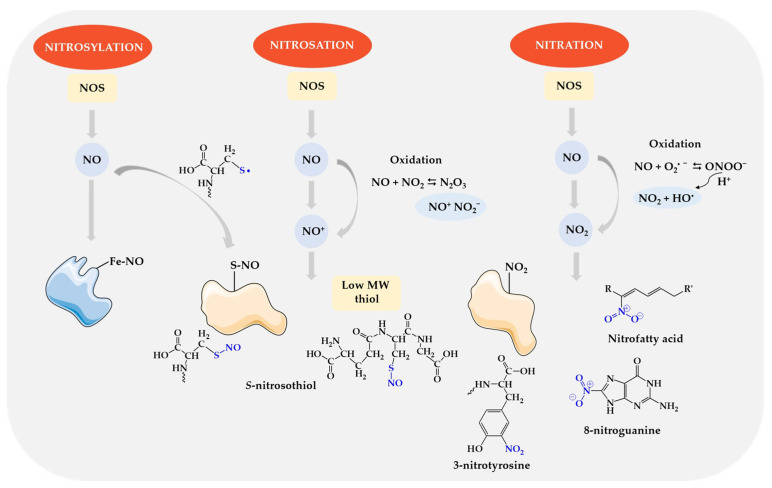
Nitrosylation, nitrosation, and nitration of biomolecules. *S*-nitrosylation involves the direct addition of NO to the reactant by coordinating NO with the metal center. Nitrosation is the reaction of a nitrosonium ion (NO^+^) with a nucleophilic group, for example, thiol residues of a protein, resulting in the formation of *S*-nitrosothiols. They can also be formed by oxidation of a thiol to a thiyl radical (RS^•^) and then direct reaction with NO. Nitration is a modification involving the attachment of a nitro group (-NO_2_) mainly to aromatic amino acids. The reaction with ONOO^−^ may produce, among others, nitrated fatty acids and 8-nitroguanine. Abbreviations: MW—molecular weight; NOS—nitric oxide synthase. This figure was created using Servier Medical Art (available at https://smart.servier.com/) (accessed on 12 January 2024).

Nitric oxide interacts with various groups of biomolecules through nitrosylation, nitrosation, and nitration [185,186]—Figure 6. These reactions may occur both in physiological conditions and in pathological conditions with disturbed redox homeostasis.

*S*-nitrosylation is the direct addition of NO to the reactant by coordinating NO with the metal center. The NO radical binds to a transition metal, e.g., heme iron (Fe), and a metal nitrosyl complex is formed. This reaction underlies the canonical activation of guanylate cyclase (sGC) by NO and the inhibition of cytochrome c oxidase (CCO) in mitochondria. Nitrosation is the reaction of a nitrosonium ion (NO^+^) with a nucleophilic group, for example, thiol residues of a protein. In the oxidation reaction, nitric oxide (II) is transformed into nitric oxide (III) (NO^+^/NO_2_^−^), which is a donor of the reactive nitrosonium cation, which is covalently attached to the sulfhydryl groups (SH) of cysteine residues, forming nitrosothiols (-SNO) [185,187]. S-nitrosothiols can also be formed by one-electron oxidation of a thiol to the thiyl radical (RS^•^) followed by direct reaction with NO. Nitration is a modification involving the attachment of a nitro group (-NO_2_) mainly to aromatic amino acids such as tyrosine, tryptophan, and histidine. Intermediate products of peroxynitrite decomposition are the main source of the nitrating agent. ONOO^−^ can also initiate free radical oxidation of lipids, which ultimately leads to the formation of lipid hydroperoxides and even nitrated fatty acids. ONOO^−^ also damages DNA, especially through a reaction with deoxyguanine, which leads to the formation of 8-oxoguanine and 8-nitroguanine [187]. Nitric oxide can also induce apoptosis directly or through ONOO^−^ [188,189].

During ischemia due to middle cerebral artery occlusion (MCAO), plasma NO concentration increases for up to 30 min and decreases in the following hours [190,191]. However, NO and peroxynitrite levels increase 4 h post-ischemia, peaking after 46–48 h, persisting for up to 7 days [192,193]. This fluctuation in NO concentration likely corresponds to different activities of NOS subtypes. Within minutes of post-MCAO induction, eNOS, and nNOS activity increases and then decreases, whereas iNOS expression is detected only after 12–70 h post-cerebral ischemia onset, persisting for about 7 days [191].

**Figure 7:**
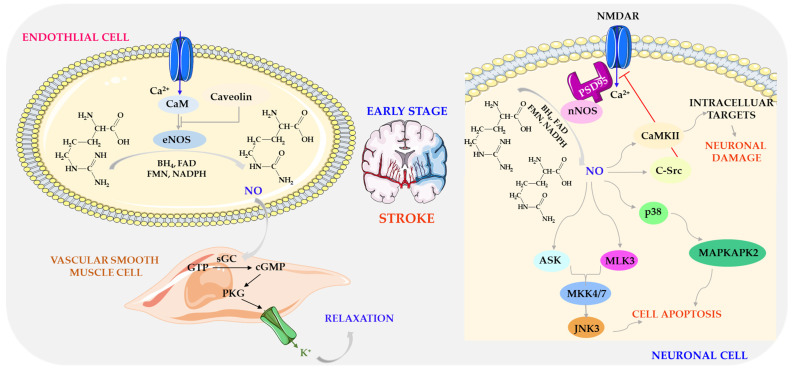
NO expression in the early stage of ischemia in stroke. eNOS increases the expression of sGC, which produces cGMP, which binds to protein kinase I (PKG I), phosphorylating the alpha subunit of the Maxi-K^+^ channel, and allowing the secretion of potassium ions (K^+^). Potassium ions hyperpolarize vascular smooth muscle cells (VSMC), causing them to relax. During brain I/R, NMDAR forms a complex with nNOS via PSD95, which ultimately leads to nNOS activation and NO synthesis. NO produced by nNOS activates c-Src to inhibit NMDAR activity. NO activates signaling molecules including ASK, MLK3, and p38, which then activate signaling proteins and ultimately stimulate the cell’s apoptosis pathway. NO also activates CaMKII through S-nitrosylation, which causes neuronal damage. Abbreviations: ASK—apoptosis signal-regulating kinase; BH_4_—tetrahydrobipterin; CaM—calmodulin-dependent protein; CaMKII—Ca^2+^/calmodulin-dependent protein kinase II; c-Scr—proto-oncogene; eNOS—endothelial NOS; FAD—flavin adenine dinucleotide; FMN—flavin mononucleotide; GTP—guanosine triphosphate; JNK3—c-jun N-terminal kinase 3; MAPKAPK2—MAP kinase-activated protein kinase 2; NADPH—nicotinamide adenine dinucleotide phosphate; NMDAR—N-methyl-D-aspartate receptor; nNOS—neuronal NOS; PSD95—protein with postsynaptic density 95; MLK3—mixed kinase 3; MKK4/7—mitogen-activated protein kinase 4/7; PKG—protein kinase G. This figure was created using Servier Medical Art (available at https://smart.servier.com/) (accessed on 12 January 2024).

The initial increase in NO concentration relates to endothelium and neuronal NO production. eNOS expression during early cerebral ischemia increases the formation of small amounts of NO, mediating vasodilation and protecting cerebral vessels [194]. This endothelial NO increases the expression of soluble guanylate cyclase (sGC), which produces cGMP. cGMP then binds to protein kinase I (PKG I), phosphorylating the alpha subunit of the Maxi-K^+^ channel, allowing potassium ion secretion, hyperpolarizing and relaxing vascular smooth muscle cells (VSMC) [195]—Figure 7.

Endothelial NO is involved in preserving and maintaining cerebral microcirculation, inhibiting platelet aggregation, leukocyte adhesion and migration, and reducing smooth muscle proliferation. NO plays a key role in regulating cerebrovascular effects by increasing or decreasing oxygen levels and elevating carbon dioxide and carbon monoxide levels and cerebrovascular autoregulation [196,197]. Decreased availability of endothelial NO plays a pivotal role in the initiation and progression of vascular diseases such as atherosclerosis. Therefore, maintaining endothelial NO production is an important strategy for preventing cerebrovascular disease [198].

eNOS-deficient mice revealed significantly impaired neovascularization after stroke, indicating that endothelium-derived NO mediates this effect [199]. Similarly, eNOS knockout mice have larger infarcts than wild-type variants after MCAO-induced ischemia–reperfusion stroke [200,201,202]. Endogenous NO produced by eNOS facilitates the expression of P-glycoprotein (P-gp), thereby protecting the BBB against ischemia–reperfusion damage in the brain [203].

In patients after ischemic stroke (IS), the level of circulating nitric oxide metabolites was assessed as nitrate/nitrite (NOx) concentration. Some studies reported lower levels of NOx [108,204,205,206], while other studies reported increased levels compared to healthy controls [207,208]. This discrepancy may be due to different NOx measurement methods or changes in the L-arginine pathway resulting from endothelial dysfunction.

Low NOx levels were associated with severe stroke (judged as the level of consciousness and total anterior circulation syndrome (TACS)) and a poor outcome was assessed as discharge disposition [204]. The increase in NOx concentration from day 1 to day 2 was associated with improved clinical outcomes in terms of neurological recovery and functional flexibility, which could be related to endothelial NOS, an isoform that has a neuroprotective effect [206]. Similarly, better results in patients with lacunar stroke are probably related to higher NO concentration in the first 24 h after cerebral infarction and lower peroxynitrite concentration [205]. However, a study by Castilo et al. found that an increase in CSF NO metabolites in AIS patients during the first 24 h was associated with greater stroke severity on admission, early neurological deterioration, larger infarct volume, and poor outcome at 3 months as assessed by CNS [208]. Similarly, Ozkul et al. found that NO levels were higher in more severe acute cerebral infarctions than in patients with less neurological deficits according to CNS scores [155].

Similarly to endothelial NO, neuronal NO appears early in ischemic stroke. As blood and oxygen supply decrease, accumulating glutamate activates calcium channels, triggering calcium-dependent nNOS to produce large amounts of NO. Excess NO modifies mixed lineage kinase 3 (MLK3) through S-nitrosylation, promoting downstream mitogen-activated protein kinase 4/7-c-jun N-terminal kinase 3 (MKK4/7-JNK) signaling pathway activation, which initiates intrinsic and extrinsic apoptosis pathways. NO also activates other signaling molecules, such as apoptosis signal-regulating kinase (ASK) and p38 protein, crucial in apoptosis control [209].

NO produced by nNOS via S-nitrosylation also inhibits the autophosphorylation of Ca^2+^/calmodulin-dependent protein (CaMKII), which causes CaMKII dysfunction and leads to brain damage [210]. However, the role of NO in neuronal damage may vary depending on the stage of cerebral ischemia and reperfusion [211]. In addition to modulating cell death-related signaling cascades, NO produced by nNOS can increase c-Src expression, inhibiting *N*-methyl-D-aspartate receptor (NMDAR) activity, leading to delayed nerve damage [212]—Figure 7.

In vivo studies suggest that NO neurotoxicity post-reperfusion and early blood–brain barrier (BBB) damage after transient focal cerebral ischemia are closely related to nNOS activity [213]. Endogenous NO produced by nNOS may contribute to BBB disruption by modulating matrix metalloproteinase (MMP) activity during cerebral ischemia/reperfusion. To initiate MMP activation, NO inhibits caveolin-1 (cav-1) expression, leading to endothelial cell hyperpermeability and BBB disruption [214].

Experimental studies using NOS inhibitors have demonstrated that NO produced by the neuronal isoform is detrimental to tissue survival and neurological outcomes [195,196,197]. Knockout studies have shown smaller infarct size and higher cerebral blood flow in nNOS-deficient mice post-cerebral ischemia–reperfusion compared to wild-type mice. Nitrate levels were also found to be significantly reduced in nNOS−/− mice [200,215,216].

**Figure 8:**
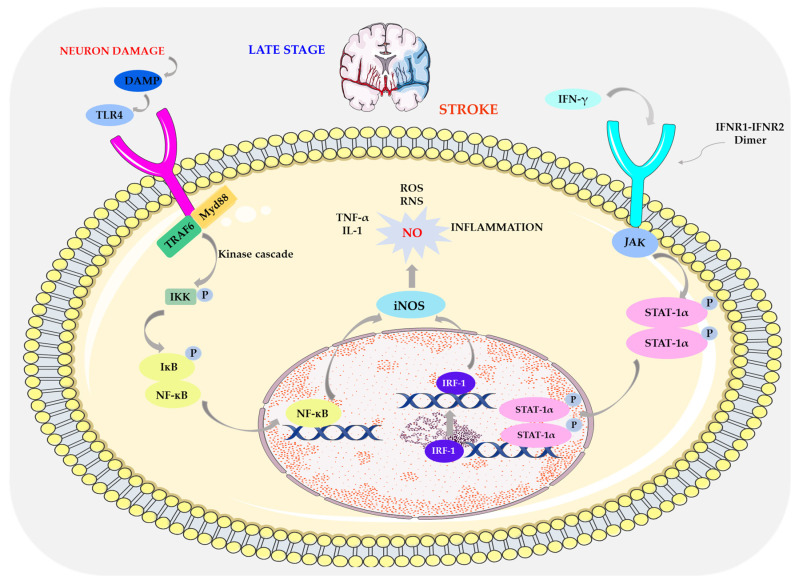
NO expression in the late stage of ischemia in stroke. Necrotic neurons release “danger signals” that activate the immune system and secrete molecular patterns (DAMPs). DAMP induces Toll-like receptors (TLRs) in microglia. The intracellular domain of the Toll/IL-1 receptor TLR4 recruits myeloid differentiation factor 88, IL-1 receptor-associated kinase, myeloid differentiation factor 88 adapter-like protein, Toll/IL-1 receptor adapter protein, and TNF receptor-related factor 6 (TRAF6) to form a complex that can further transmit the signal. Several signaling pathways are activated sequentially, including the mitogen-activated protein kinase (MAPK) pathway and the nuclear factor-κB (NF-κB) pathway, leading to phosphorylation of inhibitor of κB kinase (IKK) and subsequent phosphorylation of IκB. After phosphorylation, IκB is ubiquitinated and degraded by the proteasome, and NF-κB is released. NF-κB then translocates, interacts with the iNOS promoter, and triggers transcription of the iNOS genes. IFN-γ causes dimerization of the IFN-γ receptor and activation of Janus kinases (JAKs, such as JAK2), which then phosphorylate STAT-1α. STAT-1α dimerizes and translocates to the nucleus, where it facilitates the synthesis of interferon regulatory factor 1 (IRF-1). IRF-1 then binds to the iNOS gene promoter and induces iNOS expression. Abbreviations: DAMP—damage-associated molecular pattern; IKK—IκB kinase; IFN-γ—interferon-γ; IFNAR—interferon-α/β receptor; IκB—inhibitor of nuclear transcription factor κB; iNOS—inducible nitric oxide synthase; IL-1 alpha—interleukin-1 alpha; IRF-1—interferon regulatory factor 1; JAK—Janus-activated kinase; Myd88—myeloid differentiation primary response 88; NF-κB—nuclear factor kappa-light-chain-enhancer of activated B cells; RNS—reactive nitrogen species; ROS—reactive oxygen species; STAT-1- signal transducer and activator of transcription 1; TLR4—Toll-like receptor 4; TNF-α—tumor necrosis factor alpha; TRAF6—tumor necrosis factor receptor-associated factor 6. This figure was created using Servier Medical Art (available at https://smart.servier.com/) (accessed on 12 January 2024).

NO from neutrophil iNOS is used in the inflammatory process and stimulation of neuronal apoptosis. The iNOS isoform is not constantly present in cells and is typically expressed by proinflammatory cytokines and/or bacterial lipopolysaccharides (LPS). Induced NO is crucial for the inflammatory response and the innate immune system. The normal brain does not express much iNOS, but after trauma, inflammatory damage, or hypoxia (such as a stroke or ischemic event), iNOS can be expressed in activated glial cells and neurons [217]. After ischemic stroke, iNOS induction typically begins around 12 h, peaks after approximately 24–48 h, and persists for 1 week [192,218]. The main source of iNOS-derived NO during ischemic stroke is neutrophils. Nitric oxide (II) derived from neutrophil iNOS is used in the inflammatory process and in the stimulation of neuronal apoptosis [219]. Inducers of iNOS expression are pro-inflammatory cytokines such as tumor necrosis factor alpha (TNF-α), interleukin-1 (IL-1), and interferon-γ (IFN-γ), which bind to receptors on the cell surface and activate kinases, leading to the phosphorylation of various intracellular proteins and subsequent activation of specific transcription factors, including nuclear factor-κB (NF-κB) transcription factors such as nuclear factor transducer 1 and activator of transcription 1α (STAT-1α). The active factors then translocate to the nucleus and induce iNOS expression [220,221]—Figure 8.

The amount of NO released by iNOS is 1000 times greater than the amount released by nNOS [221,222]. NO induced by iNOS leads to brain damage during ischemia reperfusion [223], and activates apoptosis in a rat model of stroke ischemia–reperfusion injury [51,224]. MCAO-induced infarction and motor deficits were less in iNOS knockout or iNOS-deficient mice than in wild-type mice [225,226]. Experimental studies have also proven that depleting the number of iNOS^+^ inflammatory cells prevents neuronal damage not only by inhibiting NO but also by suppressing the inflammatory response because iNOS^+^ are an important source of pro-inflammatory cytokines [227]. Moreover, in patients after ischemic stroke, elevated levels of NO secondary metabolites assessed over a 2- to 7-day period predicted an increase in infarct volume, which is likely the result of activation of the inducible NOS isoform [186]. In non-lacunar stroke, high plasma nitric oxide levels 30 days after cerebral infarction were also associated with poor outcomes. A 10-unit increase in NO concentration predicted a 1-point reduction in the NIHSS neurological score [205].

After ischemic stroke, iNOS produces large amounts of NO, leading to brain damage and secondary inflammatory response induction. Elevated levels of NO secondary metabolites post-ischemic stroke predict increased infarct volume, likely due to iNOS activation [228,229]. Overexpression of iNOS promotes the secretion of proinflammatory cytokines and induces a secondary inflammatory response and free oxygen radical production [230,231]. Ischemic brain damage involves complex interactions between leukocytes, glia, neurons, and endothelium, resulting in continuous iNOS production and subsequent reactive oxygen/nitrogen species (ROS/RNS) generation [32].

During ischemia/reperfusion, NO indirectly via peroxynitrite can exert a strong cytotoxic effect, mediating neuronal cell death, inflammation, BBB disruption, and hemorrhagic transformation [232,233,234]. Additionally, peroxynitrite initiates the PINK/Parkin signaling pathway and participates in mitophagy at the reperfusion stage, contributing to brain damage [235,236]. Oxidative stress caused by increased peroxynitrite production can oxidize the BH_4_, an essential cofactor required for eNOS activity. If the BH_4_ concentration becomes suboptimal, the eNOS dimer will dissociate from other eNOS monomers, resulting in decreased endothelial NO production and increased production of superoxide anions and peroxynitrite [237].

The value of nitric oxide metabolite concentrations in human stroke is presented in Table 6.

### 7.3. Carbonyl Groups as a Result of Protein Damage in Stroke

Carbonyl groups (CG) are functional groups composed of a carbon atom connected to an oxygen atom by a double bond. They are most often formed as a result of the oxidation of protein amino acid residues.

As a result of the interaction of ROS, mainly the hydroxyl radical, with the side chains of amino acids, the peptide bond of the polypeptide chain may be broken, resulting in the formation of carbonyl groups. This process begins with the detachment of a hydrogen atom at the α carbon of an amino acid, which leads to the formation of an alkyl radical, which in the next stage reacts with oxygen to form an alkyl peroxide radical, which turns into an alkyl hydroperoxide. As a consequence, the formed alkoxy radical can be transformed into an amino acid residue hydroxylated at the α-carbon. It can also lead to α-amide fragmentation of the polypeptide chain and the formation of an *N*-α-ketoacyl derivative. Cleavage of the peptide bond can also occur as a result of the reaction of the hydroxyl radical with glutamyl and aspartyl residues of the protein polypeptide chain, which allows obtaining an N-α-acylated derivative of pyruvic acid [238].

**Figure 9:**
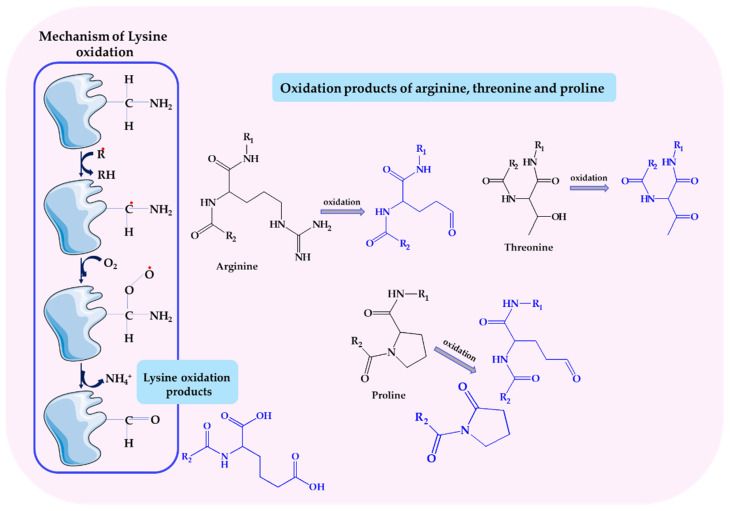
Oxidation products of amino acid residues of proteins. Oxidation of lysine leads to aminoadipic semialdehyde and 2-aminoadipic acid, proline to glutamic semialdehyde and 2-pyrrolidone, arginine to glutamic semialdehyde, and threonine residues to 2-amino-3-ketobutyric acid.

Carbonyl groups under the influence of a hydroxyl radical and in a reaction catalyzed by metal ions may also appear as a result of the oxidation of amino acid residues such as lysine, arginine, proline, and threonine (Figure 9). Oxidation of lysine leads to aminoadipic semialdehyde and 2-aminoadipic acid, proline to glutamic semialdehyde and 2-pyrrolidone, arginine to glutamic semialdehyde, and the threonine residue to 2-amino-3-ketobutyric acid. These derivatives have the ability to react with free amino groups of other amino acids in another or the same protein molecule, which may lead to the formation of cross-links [239].

Carbonyl groups are a potential marker that can be used to analyze the degree of damage to proteins and other components of a cell exposed to oxidative stress. Their concentration may reflect the degree of advancement of oxidative damage to proteins and other biomolecules [240].

The accumulation of carbonylated biomarkers in the cell is toxic and may induce a generalized autoimmune response in response to the developing inflammation [241].

There is also a disruption in the functioning of the cell’s components, which may result in the cell entering the apoptosis pathway and ultimately leading to its death.

During an ischemic stroke, large amounts of ROS are released and, as a consequence, oxidative damage to the polypeptide chain of proteins occurs, both in terms of structure and function. Proteins damaged in this way do not perform their functions properly in the cell [242,243].

There is a lack of research on protein carbonyls in AIS in the literature and, we have little information on the dynamic changes in this biomarker. Most studies are cross-sectional or have a short follow-up period after stroke. There are only a few studies on protein oxidation in AIS patients that report that protein carbonyls remained unchanged [108] or their concentration increased [5,244,245,246,247].

The authors assessed the degree of protein damage during “onset-to-needle” and after 24 h [5,224,225,226,227]. This is confirmed by the severity of oxidative stress and massive ROS production in ischemic stroke. They prove that this marker may be a progressing factor in the treatment of both AIS and hemorrhagic stroke [5,244].

There are few studies describing the contribution of carbonyl groups to the prediction of functional outcomes. A correlation was found between the level of carbonyl groups measured at the time of illness and the NIHSS scale at admission and discharge, as well as the mRS scale assessed also at admission and discharge, but also in the third month and one year after the stroke. Carbonyl groups seem to be a particularly promising biomarker because they can be considered a potential marker of the extent of hyperacute brain damage and its ultimate clinical consequences [5].

Other authors have so far managed to assess the relationship between the level of carbonyl groups and the scale of geriatric depression [225]. Post-stroke depression is a factor that adversely affects the level of oxidative damage to proteins plasma, during stroke. It is associated with increased disability and greater cognitive impairment, which leads to a delay in the rehabilitation process. Scientific research has also proven not only a significant decrease in this biomarker after post-stroke rehabilitation, but also that the extent of oxidative damage, and therefore also the level of carbonyl groups, is correlated with the state of post-stroke depression [245].

A positive correlation of carbonyl groups with an increase in the volume of the lesion, described on the basis of diffusion magnetic resonance imaging was also found [247].

The level of changes in the concentration of carbonyl groups is correlated with the degree of brain tissue damage, which then translates into the degree of physical and mental disability of stroke patients. For this reason, carbonyl groups can be used as a marker for non-invasive testing of the degree of oxidative damage, as well as for assessing the course of stroke rehabilitation and overall improvement of the patient’s condition [245].

Table 7 shows the value of CG concentrations in human stroke.

## 8. Antioxidant Therapies in Ischemic Stroke

### 8.1. Stroke Treatment

Stroke treatment has undergone a revolutionary transformation in recent years, from conservative to interventional treatment. The pharmacological treatment uses aspirin as a drug used in the acute phase and secondary prevention of cerebral infarction at a dose of 160–300 mg within 24–48 h of the onset of cerebral infarction. The effectiveness of this drug has been confirmed in clinical trials, which show that aspirin reduced the risk of serious cardiovascular events in both women and men and reduced the risk of stroke and ischemic stroke in women, without a significant increase in the risk of hemorrhagic stroke [248].

Currently, in addition to pharmacological treatment, reperfusion treatment is used: intravenous thrombolysis and mechanical thrombectomy, methods that are intended to restore impaired cerebral blood flow.

Thrombolysis involves intravenous administration of recombinant tissue plasminogen activator (rt-PA) at a dose of 0.9 mg per kilogram of body weight, up to a maximum of 90 mg. Thrombolysis is used in a very narrow therapeutic window, therefore the basic parameter determining the effectiveness of treatment is time (time is the brain). Treatment with this method can be started within 4.5 h and in special cases 6 h, from the onset of symptoms [249,250]. Patients are qualified based on the clinical diagnosis of cerebral infarction after CT (computed tomography) or MRI (magnetic resonance imaging) imaging tests. Recanalization using rtPA is effective, but there is a risk of hemorrhage, which may lead to serious consequences [251].

However, the main approach to the interventional treatment of ischemic stroke is mechanical thrombectomy, which is a procedure for patients with occlusion of large arteries and involves removing the thrombus using a microcatheter. Removal of the thrombus from an arterial vessel is performed by aspiration, using a stent-retriever or a combined technique. Compared to rtPA treatment, thrombectomy can be performed within a wider time window of 6 to 24 h after stroke onset [4].

Before the procedure is performed, after clinical verification, each patient requires computed tomography of the head and/or magnetic resonance imaging of the head with the option of an angiogram, which shows arterial vessels. In cases of occlusion of large vessels, the combination of intravenous thrombolysis and mechanical thrombectomy gives a much greater chance of treatment success [249,250].

### 8.2. Strategies to Protect the Brain against Oxidative Stress

Under physiological conditions, the production of ROS/RNS is balanced by endogenous antioxidant mechanisms. During a stroke, natural defense mechanisms begin to fail. One strategy to protect the brain against oxidative stress is the use of antioxidants, which can scavenge free radicals, inhibit the production of free radicals, or enhance their degradation [252,253].

#### 8.2.1. Antioxidants in Stroke

One of the drugs used in stroke treatment, especially in Japan and China, belonging to synthetic free radical scavengers is Edaravone (Radicut). Edaravone removes HO^•^, NO, and ONOO^−^ in a concentration-dependent manner [254,255,256]. It has an inhibitory effect on lipid peroxidation in the arachidonate cascade, and can also inhibit the inflammatory response during ischemia–reperfusion [257,258,259]. In an animal experiment, Edaravone increased the release of NO produced by eNOS [260]. The effectiveness of this drug ranges from major clinical improvement to moderate effects as measured by standard stroke scales when administered up to 72 h after ischemic stroke [261,262].

In experimental studies, treatment with a peroxynitrite decomposition catalyst (PDC) in rodent brain I/R models showed a reduction in BBB damage and neurological dysfunction [234]. Moreover, after PDC treatment at the reperfusion stage, inhibition of dynamin-related protein 1 (Drp1) and Parkin translocation was observed, accompanied by attenuation of cerebral infarcts and neuronal damage [263].

Another free radical scavenger is naturally occurring uric acid. It removes hydroxyl radicals, hydrogen peroxide, and ONOO^−^, inhibits the Fenton reaction, and increases SOD 3 activity [248,264]. In people treated with intravenous thrombolysis, it has a neuroprotective function due to its antioxidant properties. High concentrations of this acid in post-stroke patients were associated with better clinical and functional outcomes [265,266,267,268]. An experiment in rats showed that uric acid injected intravenously in combination with tPA reduced infarct volume and resulted in better neurological function compared to tPA alone [269].

Supplementation with the antioxidant vitamin C, which directly removes ROS and RNS as well as increases NO synthesis through eNOS, may reduce the risk of ischemic and hemorrhagic stroke and lipid peroxidation [6,262,270]. Vitamin E reduces infarct volume by 45–55% in transient and permanent cerebral ischemia models [271,272].

#### 8.2.2. ROS/RNS Inhibitors in Stroke

Inhibition of enzymes generating reactive oxygen and nitrogen species, especially NADPH oxidase (NOX), xanthine oxidase (XO), and myeloperoxidase, is another approach to reducing oxidative stress in stroke [253].

Inhibition of NOX may be used as a target in the treatment of stroke, especially NOX2 and NOX4 may be targets for cytoprotective therapies after ischemia. The most popular NOX inhibitor in experimental stroke is apocynin, which inhibits superoxide release from NOX by blocking the migration of p47phox to the membrane, thereby interfering with the formation of a functional NOX complex [273,274]. Other natural compounds of NOX inhibitors are honokiol and plumbagin [273]. VAS2870 is a pharmacological inhibitor of NOX and has been shown to reduce infarct volume, oxidative stress, neuronal apoptosis, and BBB leakage [253].

Allopurinol, an XO inhibitor, reduces infarct volume by 32–35% and the formation of cerebral edema and improves the functioning of the blood-brain barrier in a rat model of ischemic stroke [275]. Caffeic acid phenethyl ester (CAPE) derivatives are also good candidates for XO inhibitors [276].

There are many active compounds with bioactive properties that inhibit MPO activity, which can be used to alleviate cerebral ischemia–reperfusion damage. This group includes some bioflavonoids such as quercetin, rutin, eriodictyol, isorhamnetin, biochanin A, baicalin, polyphenols, for example, resveratrol, curcumin or cannabidiol, and compounds belonging to alkaloids, saponins, terpenoids, and coumarins [277].

Inhibiting factors contributing to the worsening of cerebral infarction may be a potential strategy for the treatment of ischemic stroke.

The key role in mitigating oxidative stress during ischemia is played by the transcription factor NF, E2-related factor 2 (Nrf2) in signaling pathways such as Keap1 proteins, PI3K/AKT, MAPK, nuclear factor kappa-light-chain-enhancer of activated B cells (NF- κB) and heme oxidoreductase-1 (HO-1). Nrf2 activation is caused by excessive ROS production after cerebral ischemia. Once activated, Nrf2 initiates the transcription of a number of antioxidant genes and attenuates blood–brain barrier disruption and inflammation [278,279]. Many therapeutic substances/drugs are used in Chinese medicine to alleviate oxidative stress during ischemia by regulating Nrf2 signaling pathways. These drugs play a protective role in ischemic stroke by regulating oxidative stress, although their specific regulatory mechanisms are different. Recently, Rutaecarpine has been tested, among others, and it turned out that it improves neuronal damage, and inhibits apoptosis, inflammation, and oxidative stress by regulating the expression of the ERK1/2 and Nrf2/HO-1 pathway in rats with cerebral ischemia–reperfusion injury [280]. Theaflavin alleviates cerebral ischemia/reperfusion injury by abolishing miRNA1283-mediated Nrf2 inhibition and reducing oxidative stress [281]. Carvacryl acetate provides neuroprotection via the Nrf2 signaling pathway [282]. Geraniin protects against cerebral ischemia/reperfusion injury by suppressing oxidative stress and neuronal apoptosis by regulating the Nrf2/HO-1 pathway [283]. Melatonin has an activating effect on Nrf2 and its target genes, increases HO-1 expression, and amelioration of brain edema, BBB impairment, apoptosis, and neurological deficits [284]. These are just some examples of naturally occurring compounds that modulate Nrf2 signaling pathways, which protect the brain against oxidative stress by reducing ROS, lipid peroxidation products (MDA), increasing antioxidant protection (increasing the expression of SOD, CAT, GPx), inhibiting ROS-generating enzymes and regulating NO synthesis [278].

Therapeutic strategies based on NO signaling modulation may mitigate the risk of serious and irreversible complications after stroke. Increasing or modulating endogenous NO production/bioavailability by activating the PI3K/Akt/eNOS signaling pathway may be a therapeutic approach in stroke. Vitexin is able to increase Akt and eNOS phosphorylation to maintain BBB integrity in an ischemic stroke model, which can be abrogated with a PI3K inhibitor [285]. CXC195, a novel tetramethylpyrazine derivative, can alleviate brain I/R damage in the same way as Vitexin [286]. Another strategy is to disrupt the NR2B-PSD95-nNOS complex. For example, one of the bioactive components of Magnolia officinalis, honokiol, reduces the interaction between nNOS and PSD95 but also inhibits the translocation of nNOS from the cytosol to the membrane, which ultimately improves the course of ischemic stroke in mice [287].

#### 8.2.3. Other Antioxidant Strategies in Stroke

Degradation of free radicals through the endogenous antioxidant mechanism may also be one of the therapeutic strategies in stroke. Experimental studies on animals have shown that SOD1 and SOD2 protect against damage caused by oxidative stress. Underexpression of SOD1 in SOD1−/− knockout mice resulted in mortality within 24 h of MCAO. In SOD1+/− mice, increased infarct volume and edema were found compared to controls [288]. Heterozygous mice (SOD2+/−) had increased levels of superoxide radical and showed an increase in infarct volume after cerebral ischemia compared to wild-type individuals [289]. Gene therapy targeting the increase in SOD1 and/or SOD2 expression may have therapeutic potential against oxidative stress in cerebral infarction [73]. The use of nanomaterials as carriers enabling the transport of antioxidant enzymes to the brain also seems to be a good solution [290]. The nanocarrier reduces enzyme proteolytic degradation, and immunogenicity, and increases its half-life and BBB permeability. For example, CL SOD1 nanozymes show a prolonged ability to scavenge experimentally induced reactive oxygen species. In vivo, they reduce ischemia/reperfusion-induced tissue damage and improve sensorimotor function in rat MCAO after a single intravenous injection [291].

In recent years, the number of preclinical studies on the use of mesenchymal stem cells (MSCs) in ischemic stroke has increased significantly. One of the important processes of MSC therapy is the secretion of paracrine exosomes by stem cells, which are characterized by low immunogenicity and the ability to cross the BBB [292,293].

MSCs promote post-stroke recovery by secreting factors that enhance brain repair and plasticity in rodents [294,295,296]. They have a neuroprotective effect by modulating the ubiquitin–proteasome system (UPS) and autophagy under cerebral ischemia [297]. The therapeutic effect of MMC is also associated with increasing the level of Miro1, which is responsible for the transport of mitochondria from mesenchymal multipotent stromal cells (MMSCs) to damaged neuronal cells after ischemia [298].

In combined therapy with mesenchymal stem cells and exosomes, not only the brain infarct surface is reduced and neurological regeneration in rats after AIS is improved, but there is also a reduction in markers of inflammation and oxidative stress such as inducible nitric oxide synthase, NOX1, NOX2 and the degree of protein oxidation [299].

Combination stem cell treatment with a tissue inhibitor of matrix metalloproteinase 1 and 2 (TIMP 1 and 2) transgene and metabolic supplementation (antioxidants and L-arginine) also seem to be beneficial. Histological and functional results after cerebral ischemia improved significantly. With homing stem cells to the brain, neurogenesis/angiogenesis, NO bioactivity, and decreased systemic oxidative stress and MMP activity were increased [300].

Combined overexpression of neuroglobin (Ngb) with inhibition of SP600125 N-Jun N-JB kinase (JNK) reduced reoxygenation-induced oxidative stress and apoptosis in cultured neurons and reduced infarct and improved neurological outcomes more than single post-stroke treatment in vivo in rats [301].

Multimodal therapy may influence the targeting of treatment for a neurological patient and extend the time window [302].

The newly developing field of proteomics plays a key role in the search for molecular biomarkers of oxidative stress. The proteome is highly dynamic and adapts in response to changes in the environment through transcriptional, translational, post-translational, and degradative processes.

mRNA regulation plays an important role in diseases and injuries and occurs in cerebral ischemia and reperfusion after stroke and in cardiac arrest and resuscitation. It has been shown that cerebral ischemia and reperfusion are accompanied by translational arrest in neurons due to stress. Post-translational protein modifications may be important modulators of stroke outcome [303].

miR-424 levels have been shown to decrease in the plasma of patients with acute ischemic stroke, therefore miR-424 treatment reduces cerebral infarct volume by increasing the expression and activation of antioxidant enzymes and Nrf2 [304].

#### 8.2.4. Potential Antioxidant Drugs in Stroke

Current therapies, thrombolysis, and thrombectomy, used in stroke, are aimed at recanalization of the occluded vessel and reperfusion of damaged brain tissue. Restoring normal levels of blood flow is crucial to their effectiveness, which is why in recent years there has been an increasing amount of research into drugs that support the above-mentioned therapies. Their main task would be to protect brain cells after ischemia.

In this context, the development of antioxidant drugs, free radical scavengers, activators of endogenous antioxidant systems, and compounds acting directly on abnormal mechanisms activating radical stress is of key importance.

In Chinese medicine, many drugs are used in complementary therapy, the main task of which is regeneration after a stroke. They may improve the quality of life after a stroke and reduce neurological deficits. These are mainly botanical medicines. They are characterized by good stability, negligible side effects, and long-term effects on ischemia. Compared to standard drugs, however, they are not homogeneous preparations, which affects their absorption and slow action. The article discusses only selected pharmaceutical preparations based on Chinese herbs that offer potential therapeutic and prophylactic use in the treatment of stroke, and which have not been approved by the European Commission at the moment.

The most commonly used plants with antioxidant therapeutic effects in stroke in Chinese folk medicine are Chuanxiong Rhizoma, Safflower, Musk, and Salvia miltiorrhiza [305].

Salvia miltiorrhiza is one of the most popular Chinese medicines, listed in the Chinese Pharmacopoeia, for the treatment of stroke. The main components of this plant are tanshinone and sage acids. It also contains baicalin, ergosterol, and ursolic acid. Among the phenolic acids, salvianic acid B is the most biologically active, preventing brain ischemia–reperfusion damage in rats by reducing free radicals, improving energy metabolism, and regional cerebral blood flow in the ischemic hemisphere [306,307]. Tanshinone effectively prevents ischemic damage to the central nervous system and protects neurons in the ischemic area [308].

Musk significantly improves behavioral outcomes and cerebral infarct volume in rats. The chemical components of musk include steroids, lipids, peptides, and macrocyclic musk compounds. The most important is muscone, which has a neuroprotective effect against stroke injuries [309].

Safflower is used to treat coronary heart disease, stroke, and hypertension, among others. Safflower components associated with stroke are hydroxysafflor yellow A, kaempferol-3-o-rutinoside (KRS), and kaempferol-3-o-glucosides (KGS) [305].

Whereas Chuanxiong rhizome is clinically used for cerebral ischemia, it has a neuroprotective effect. Ferulic acid and ligustrazine are mainly responsible for its therapeutic effect [310].

Recently, the effectiveness of other plant and mushroom substances such as Cro-cetin from Crocus sativus, Nigella Sativa and its component Thymoquinone, Scutellaria bacalensis, Ecdysterone from Achyranthes bidentata Blume, Notoginseng leaf, Crocus sativus L., Vialinin A has also been assessed in the treatment of stroke [311,312,313,314,315,316,317]. They can inhibit the activation of NADPH oxidase 2 (NOX2) to reduce reactive oxygen species (ROS) and PAR production in the early stage of parthanatos [311], have neuro-protective effects [312,313], alleviate ischemic brain damage [318], improve oxidative damage in rats MCAO by inhibiting ferroptosis [315], inhibit mitochondrial damage and alleviate the dysfunction of energy metabolism [316], scavenge free radicals [317].

Chinese medicine also uses chemical compounds isolated from natural substances, which can be treated as potentially effective drugs with antioxidant properties in the treatment of ischemic stroke. This group of compounds includes 3-N-butylphthalide (NBP), scutellarin, caffeic acid (3,4-dihydroxycinnamic acid, CA), and salidroside. They may have protective effects during ischemia [319,320], directly scavenge free radicals and NO during oxidative damage [321], induce SOD, GSH-Px, and GST activity in the MCAO model, significantly alleviating brain I/R damage [322].

Traditional Chinese patent medicines prepared under the guidance of TCM theory, which are not yet approved for marketing in European Union countries, can be considered potential stroke medicines. Of these, the Angong Niuhuang, Qishiwei Zhen-zhu, Ginkgo biloba leaf, Huatuo Zaizao, Xueshuan Xinmai Ning pills are worthy of note [305].

These drugs have a rich herbal composition and great potential in the treatment of ischemia, improve the effectiveness of stroke treatment, increase SOD activity in the hippocampus, reduce the level of MDA, and increase cerebral blood flow and blood supply to the cardiovascular system [305,323].

In addition to a huge number of potential drugs of natural origin, attention should also be paid to synthesized chemical compounds belonging to the so-called radical traps, i.e., N-oxides of the imine group or nitrones. They enable the detection of unstable and short-lived free radicals in electronic paramagnetic resonance (EPR) techniques by creating products with longer half-lives [318].

One of them, NXY-059 (Cerovive, OKN-007), a derivative of phenyl-N-tert-butylnitrone (PBN), reached phase III clinical trials, but due to low permeability profile and systematic failures in vivo experimental tests, the studies were discontinued [324].

This group includes TBN (tetramethylpyrazine nitron), derived from the active ingredient of the traditional Chinese herb Ligusticum wallichii, which has strong neuroprotective properties in ischemic stroke, combining a thrombolytic effect with strong radical scavenging properties [325,326].

Based on TBN, compounds such as TN-2 [327] and CT-011 [328] have been developed, which have also proven to be effective in various experimental models of ischemic stroke.

Nitrones based on quinoline and cholesterol are suitable candidates for ischemic stroke drugs. Among the tested compounds, the best results were achieved by quino-thylnitron QN23 and cholesteronitron ISQ201. In preclinical studies on animal models, their neuroprotective effect was confirmed even up to several hours after the start of reperfusion [318].

A promising group of compounds may also be chalcones, which come from the flavonoid family. Synthetic (E)-3,4-dihydroxychalcone analogs can be used as anti-ischemic stroke agents that attenuate oxidative stress by directly scavenging ROS and activating the Keap1/NRF2/ARE pathway. The electrophilic α, β-unsaturated ketone moiety (Michael acceptor) on chalcone is responsible for the dual antioxidant properties [329].

Potential drug candidates discussed cover a wide range of applications, from herbal medicines popular in China to synthetic nitrones and chalcones.

When choosing such drugs, prior clinical assessment should always be taken into account. It is important to use them responsibly to achieve the best results and enjoy the highest level of health.

### 8.3. Stroke Prevention

Despite increasing therapeutic options and an increasing level of knowledge, stroke treatment is still a major challenge and the leading cause of disability in the world. It has a socio-economic impact, as one-third of all stroke patients require ongoing inpatient care. For this reason, according to the principle that prevention is better than cure, it seems that prevention is the best strategy for preventing this disease and should be treated equally with the causal treatment.

Risk factors for stroke can vary, ranging from non-modifiable factors such as age, gender, race, and genetic predispositions to be modifiable, including vascular and behavioral. According to research, 85% of strokes can be prevented because most of the factors predisposing to stroke are modifiable [330].

The main modifiable risk factor for stroke is hypertension. It is estimated that half of strokes are the result of uncontrolled hypertension [330]. Even reducing systolic blood pressure by 2 mmHg reduces the risk by 25% and reduces diastolic blood pressure by even 50% [331].

An increase in hypertension also poses a serious risk of recurrent stroke [332].

The most important cardiological causes of stroke include atrial fibrillation, atherosclerosis of intracerebral and extracerebral arteries, rheumatic heart disease, and myocarditis. Each of these diseases increases the risk by 3.5 times [330].

The remaining factors subject to regulation are the main components of the so-called metabolic syndrome (MetS), which generally refers to metabolic abnormalities including: (i) central obesity (ii) atherogenic dyslipidemia (iii) hyperglycemia. The metabolic syndrome also includes the previously mentioned hypertension. Factors of the metabolic syndrome increase the risk of vascular disease, promoting the development of atherosclerosis and type II diabetes 2 [333]. As the number of MetS components increases, the risk of incident ischemic stroke increases; in prospective studies, the hazard ratios for incident ischemic stroke associated with MetS range between 2.1 and 2.47, and a hazard ratio as high as 5.15 [334,335].

The main cause of metabolic abnormalities may be insulin resistance (IR), which, together with central obesity, may contribute to the development of MetS [333,336,337].

IR and obesity may therefore be independent risk factors for ischemic stroke. IR disturbs the metabolism of glucose, lipids, and proteins; therefore, it accelerates the development of type 2 diabetes, and thrombosis and promotes the development of atherosclerosis. It affects the intensification of ROS production, resulting in an increase in the concentration of free fatty acids, promoting glucotoxicity, lipotoxicity, and translocation of the nuclear factor NF-kB (87) and pro-inflammatory signaling [338,339].

Metabolic disturbances accompanying peripheral IR are associated with poor prognosis in patients with ischemic stroke, exacerbating the inflammatory response, oxidative stress, and neuronal damage [340].

Obesity (especially visceral obesity) initiates a cascade of reactions resulting in metabolic dysfunctions in the form of insulin resistance and hyperinsulinism, as well as abnormalities in plasma lipid concentrations [341].

The risk of stroke decreases by up to 25–30% with weight loss [342].

Lifestyle changes, including a healthy diet, weight loss, smoking cessation, and adequate physical activity, are well-known ways to improve peripheral insulin sensitivity and are considered primary prevention of ischemic stroke. Similarly, reducing abdominal obesity by increasing physical activity may have positive benefits in reducing the risk of stroke. Appropriate prevention in the form of a healthy lifestyle reduces the incidence of hypertension, hyperlipidemia, obesity, diabetes, atherosclerosis, and other cardiovascular diseases that predispose to stroke.

### 8.4. Future Prospects in Stroke Treatment

Despite numerous studies at the molecular level, stroke remains one of the main causes of death and disability in patients, with limited therapeutic, diagnostic, and prognostic possibilities. In recent years, much research has been carried out aimed at preventing and treating stroke. However, so far, no biomarker has been found to support the patient’s diagnosis/prognosis or is necessary for the development of therapies to counteract the harmful effects of its action [343].

It is known that oxidative stress plays a key role in the mechanisms of cerebral ischemia–reperfusion injury, therefore a better understanding of them may allow the development of rational therapeutic approaches. However, it should be noted that oxidative stress is associated with many diseases with energy metabolism disorders, neuroinflammation, and tissue damage, therefore diagnosing ischemic stroke using oxidative stress markers is quite a challenge [87]. Research to date is inconclusive, and substances with antioxidant activity are still not recommended in the treatment of the acute phase of ischemic stroke. This may be due to the difficulties in planning and testing many thousands of animals and people, the duration of experiments, and financial outlays.

The use of multi-oxidant therapy with antioxidant molecules such as polyphenols, carotenoids, vitamins, and others with a broad spectrum of action may alleviate damage after I/R, participating in multiple signaling pathways at different stages of stroke, assist in monitoring the severity of ischemic stroke and patient prognosis.

Currently, high hopes are associated with, among others, uric acid and Edaravone. The attractiveness of the hypothesis about the neuroprotective effect of drugs from this group and the experience gathered so far are the basis for further research.

Antioxidant nanoparticles can be used to enhance the in vivo effectiveness of traditional antioxidant substances. Nanoparticles provide antioxidant/enzyme protection while effectively delivering them to inaccessible areas such as the brain. They are able to cross the blood–brain barrier and easily reach ischemic areas [344].

MSC combination therapy may be a promising tool, providing patients with better functional outcomes.

Strengthening the antioxidant defense may be one of the most important strategies in the treatment of impaired redox homeostasis in stroke through an appropriate diet rich in antioxidants and pharmacological intervention.

A promising new technology is proteomics, which, by generating experimental and clinical data, can support the search for molecular markers of oxidative stress in stroke [345]. Stroke proteomics may therefore hold powerful promise for use in stroke diagnosis and prevention [343]. However, developing artificial intelligence methods may be helpful in their search in the future.

In order for compounds with antioxidant activity or oxidative stress markers to become part of the standard of care in the treatment of ischemic stroke, experimental and clinical research is necessary, conducted according to the modern Stroke Therapy Academic Industry Roundtable (STAIR) criteria.

The focus should be on pleiotropic, multitarget factors that can intervene at multiple levels of the ischemic cascade. Combination therapies may provide many benefits, combining antioxidants with existing thrombolytics or new neuroprotectants may represent an avenue worthy of clinical trials. We should also not forget about prevention. In order to avoid and alleviate the drastic effects of ischemic stroke, you should change your lifestyle, take care of your diet, and exercise to strengthen the antioxidant barrier.

## 9. Conclusions

In the presented review, we tried to present the most important information related to oxidative stress, mechanisms of ROS formation, and the role of the antioxidant barrier in stroke. The information contained in the article includes studies assessing the concentration of oxidative stress biomarkers and the level of antioxidants and their impact on the clinical prognosis of patients after stroke.

We also drew attention to the role of new antioxidant therapies in the fight against redox imbalance in order, to reduce brain damage caused by both ischemia and reperfusion.

## Figures and Tables

**Table 1:** The value of TAC concentration in human stroke.

Biomarker /Unit		Biological Material /Collection Time (after Stroke)		Average Concentration	Control	Reference
**TAC**	[µmol/L]	Serum	24 h	**5.72 ± 1.41** *** (n = 100)	**8.53 ± 2.41** (n = 99)	[88]
			30 days	**0.65 ± 0.08** * (n = 14)	**1.31 ± 0.21** (n = 15)	[77]
			Less than 48 h	**1043.4 ± 140.7** (n = 73)	**1079.7 ± 197.9** (n = 62)	[92]
			24 h	**540 (38)** (n = 31)	**550 (38)** (n = 26)	[91]
			48–72 h	**516 (28)** (n = 31)	**545 (70)** (n = 26)	
			7 days	**487 (39)** (n = 31)	**603 (113)** (n = 26)	
			48 h	**3687.35** (801.56) (n = 216)	**3905.98 (879.89)** (n = 188)	[90]
	mmol/L		1 day	**1.7 ± 0.30** *** (n = 60)	**4.20 ± 0.50** (n = 30)	[93]
			48 h	**1.41 (0.13)** (n = 35)	**1.39 (0.15)** (n = 44)	[96]
	mM		24 h	**10.03 ± 1.97** *** (n = 29 diabetic patients) **5.97 ± 2.04** *** (n = 36 nondiabetic patients)	**5.44 ± 1.06** (n = 20)	[94]
	mmol/mL		1 day	**2.38 (1.83–3.35)** ** (n = 29 survivors) **5.33 (3.27–11.40)** (n = 29 non-survivors)	**-**	[84]

* *p* < 0.05, ** *p* < 0.01, *** *p* < 0.001 differences statistically significant with control, “-”—no data

**Table 2:** The value of GPx, CAT, and SOD activity in human stroke.

Biomarker /Unit		Biological Material /Collection Time (after Stroke)		Average Concentration/Activity Value of Biomarker in Human Stroke		Control	Reference
**GPx**	U/g Hb	Hemolysate	12 h	**23.6 ± 7.34** *** (n = 40)		**77.7 ± 22.6** (n = 35)	[111]
				NIHSS ≤ 10	29.1 ± 11.3 (n = 23)		
				NIHSS > 10	16.7 ± 8.5 (n = 17)		
				mRS ≤ 3	26.8 ± 12 (n = 22)		
				mRS > 3	19.2 ± 19 (n = 18)		
			24 h	**26.2 ± 5.1** *	(n = 34)	**30.5 ± 7.9** (n = 30)	[114]
			7 days	**32.4 ± 4.6**			
			24 h	**36.16 ± 4.91** *	(n = 82)	**24.83 ± 1.24** (n = 81) **3.61 ± 0.17**	[109]
			7 days	**26.28 ± 1.51**			
			3 months	**23.55 ± 2.60**			
**CAT**	µkat/g Hb		24 h	**4.92 ± 0.17** ***			
			7 days	**6.56 ± 0.24** ***			
			3 months	**4.85 ± 0.21** ***			
**GPx**	nmol/min per mg of proteins		-	(n = 75)		**27.4 ± 3.52** (n = 23)	[113]
				NIHSS 4.6 ± 0.5, n = 9	**36.2 ± 6.13**		
				NIHSS 8.9 ± 0.7, n = 32	**35.2 ± 3.11**		
				NIHSS 15.4 ± 1.8, n = 34	44.2 ± 5.50 *		
		Plasma		(n = 75)		**2.64 ± 0.33** (n = 23)	
				NIHSS 4.6 ± 0.5, n = 9	**6.77 ± 1.31** **		
				NIHSS 8.9 ± 0.7, n = 32	**4.40 ± 0.68** *		
				NIHSS 15.4 ± 1.8, n = 34	**9.49 ± 1.67** ***		
	U/L	Serum	6 h	**128 ± 44**	(n = 11)	**113 ± 36** (n = 17)	[112]
			1 day	153 ± 37 *			
			3 days	**165 ± 55**			
			7 days	**147 ± 61**			
**SOD**	U/mL		Admission (8–12 a.m.)	**4.5 ± 0.7** *** (n = 35)		**3.4 ± 0.6** (n = 32)	[108]
	U/mg Hb	Hemolysate	24 h	**697.62 ± 8.95** ** (n = 82)		**661.44 ± 7.15** (n = 81)	[109]
			7 days	**750.45 ± 9.72** *** (n = 82)			
			3 months	**747.41 ± 17.27** ** (n = 82)			
	U/gHb		12 h	NIHSS ≤ 10	**1.75 ± 0.5** (n = 23)	Figure 1c in [111]	[111]
				NIHSS > 10	**2.3 ± 0.3** (n = 17)		
				mRS ≤ 3	**1.8 ± 0.3** (n = 22)		
				mRS > 3	**2.2 ± 0.4** (n = 18)		
	U/L	Plasma		NIHSS ≤ 10	**1.8 ± 0.3** (n = 23)	Figure 2b in [111]	
				NIHSS > 10	**2.2 ± 0.4** (n = 17)		
				mRS ≤ 3	**1.8 ± 0.5** (n = 22)		
				mRS > 3	**2.1 ± 0.4** (n = 18)		
	U/µmol Hb	Hemolysate	Admission	**12.2 ± 2.9** (n = 11)		**15–22** (n = 101)	[112]
			1 day	**12.7 ± 4.3** (n = 11)			
			3 days	**10.7 ± 1.2** (n = 11)			
			7 days	**12.8 ± 2.6** (n = 11)			

* *p* < 0.05, ** *p* < 0.01, *** *p* < 0.001 differences statistically significant with control; “-”—no data

**Table 3:** The value of MEL concentrations in human stroke.

Biomarker /Unit		Biological Material /Collection Time (after Stroke)		Average Concentration	Control	Reference
**MEL**	[pg/mL]	Urine	4.5 h	**6.41 [4.26, 8.64]** *** (n = 77)	**37.85 [21.46, 103.82]** (n = 23)	[5]
			24 h	**9.34 [5.49, 25.42]** *** (n = 44)		
			7 days	**9.61 [6.04, 15.33]** *** (n = 31)		

*** *p* < 0.001 differences statistically significant with control.

**Table 4:** The average GSH concentration values in human stroke.

Biomarker /Unit		Biological Material /Collection Time (after Stroke)		Average Concentration		Control	Reference
**GSH**	µM	Plasma	10–24 h	NIHSS > 10 **GSH ≤ 1.07 μM vs. GSH > 2.64 μM** (n = 43)		-	[151]
				Age/gender-adjusted odds ratio: 4.69, 95% CI: 1.43–15.4			
	mg/dL	Serum	48 h	**3.9 ± 2.5** * (n = 70) 95% CI: 0.99–2.19		**2.3 ± 0.4** (n = 70)	[155]
				Lower CNS Higher CNS	**5.37 ± 2.8** (n = 6) **3.85 ± 2.4** (n = 64)		
	µM		Admission	**84 ± 26** * (n = 11)		**68 ± 97** (n = 101)	[112]
			Day 1	**78 ± 39** (n = 11)			
			Day 3	**81 ± 42** (n = 11)			
			Day 7	**77 ± 34** (n = 11)			

CNS—Canadian Neurological Scale; * *p* < 0.05 differences statistically significant with control; “-”—no data

**Table 5:** The average MDA concentration values in human stroke.

Biomarker /Unit		Biological Material /Collection Time (after Stroke)		Average Concentration		Control	Reference
**MDA**	mol/gHb	erythrocyte	24 h	**349.7 ± 60.5** *** (n = 34)		**200.0 ± 605.7** (n = 30)	[114]
			After 7 days	**80.5 ± 47.3–269.2 ± 40.9** (n = 34)			
	µmol/L	Plasma	12 h	NIHSS ≤ 10	**17.1 ± 3.5** (n = 23)	Figure 2d in [111]	[111]
				NIHSS > 10	**18.6 ± 5.4** (n = 17)		
				mRS ≤ 3	**17.4 ± 7** (n = 22)		
				mRS > 3	**18.9 ± 6.5** (n = 18)		
	nmol/g protein	Serum	Admission (8–12 a.m.)	**151.5 ± 51.1** *** (n = 35)		**111.3 ± 27.4** (n = 32)	[108]
	µmol/L		24–72 h	**5.10 ± 1.26** (n = 60)		**3.27 ± 0.52** (n = 30)	[164]
			24 h	**7.11 ± 1.67** (n = 100)		**1.64 ± 0.82** (n = 99)	[88]
			48 h	**2.08** (n = 216)		**1.85** (n = 188)	[90]
			0 h	**0.75 ± 0.06** (n = 45)		**0.83 ± 0.06** (n = 45)	[168]
			48 h	**1.65 ± 0.08** (n = 45)			
			-	**2.51 ± 1.11** (n = 50)		**1.12 ± 0.35** (n = 25)	[170]
	nmol/L		0 h	**8.47 ± 2.46** (n = 20)		**5.99 ± 2.28** (n = 27)	[166]
			24 h	**9.75 ± 1.94** (n = 20)			
			48 h	**8.17 ± 2.14** (n = 20)			
			72 h	**8.39 ± 3.13** (n = 20)			
			96 h	**7.93 ± 2.35** (n = 20)			
	µM/mg protein		8 h	**1.433 mild AIS** (n = 85) **1.505 moderate AIS** (n = 37)		1.526 (n = 40)	[167]
	µmol/L	Saliva	-	**0.64 ± 0.22** (n = 50)		**0.23 ± 0.07** (n = 25)	[170]

*** *p* < 0.001 differences statistically significant with control; “-”—no data

**Table 6:** The value of nitric oxide metabolites concentrations in human stroke.

Biomarker /Unit		Biological Material /Collection Time (after Stroke)		Average Concentration		Control	Reference
**NOx**	µmol/L	Serum	48 h	**35.1 ± 12** * (n = 70)		**14.9 ± 3.8** n = 70	[155]
				Lower CNS	**67.9 ± 12.04** * (n = 6)		
				Higher CNS	**39.35 ± 24.5** * (n = 64)		
			-	**76.4 ± 53.3** *** (n = 54)		**41.5 ± 27.0** (n = 50)	[207]
			24 h	No infarct volume growth	**9.3 (8–14.6)** (n = 44)	15.3 (12.1–17.9) (n = 14)	[206]
				Infarct volume growth	**9.1 (5.9–14.4)** (n = 26)		
			48 h	No infarct volume growth	**10.6 (7.6–13.6)** (n = 44)		
				Infarct volume growth	**9.9 (6.9–14)** (n = 26)		
			Increase (day 7-day 1)	No infarct volume growth	**5 (1.9–12.2)** (n = 44) *		
				Infarct volume growth	**1.2 (0–7.6)** (n = 26) *		
	µmol/g protein		Admission (8–12 a.m.)	**12.4 ± 6.8** *** (n = 35)		**17.4 ± 2.5** (n = 32)	[108]
	nmol/mg protein	Plasma	24 h	**51.10 ± 12.50** *** (n = 47)		**115.40 ± 12.40** (n = 30)	[205]
				Non-lacunar	**41.8** (9.3; n = 23) ***		
				lacunar	**60.0** (7.9; n = 24) ***		

* *p* < 0.05, *** *p* < 0.001 differences statistically significant with control; “-”—no data

**Table 7:** The value of CG concentrations in human stroke.

Biomarker /Unit		Biological Material /Collection Time (after Stroke)		Average Concentration	Control	Reference
**CG**	U/mL	Serum	<4.5 h	**222 [171, 283]** ** (n = 81)	**144 [93, 191]** (n = 22)	[5]
			24 h	**355 [224, 437]** ** (n = 37)		
			192 h	**195 [124, 242]** * (n = 34)		
	nmol/mg protein		24 h	**4.9744 ± 5.2691** *** (n = 18)	**1.28.3 ± 39.3** (n = 34)	[244]
			Admission (7–9 a.m.)	**105.32** *** (n = 31)	**41.56** (n = 20)	[245]
		Plasma	Admission Discharge	**1.21 ± 0.24** (n = 20) * 0.79 ± 0.16 (n = 20) *	**0.70 ± 0.12** (n = 24)	[246]
			24 h 192 h 3 months	**0.22 ± 0.03** (n = 82) **0.28 ± 0.04** (n = 82) **0.20 ± 0.07** (n= 82)	**0.25 ± 0.04** (n = 81)	[109]

* *p* < 0.05, ** *p* < 0.01, *** *p* < 0.001 differences statistically significant with control.
